# Guideline for secondary use of health records within Norwegian and EU regulatory frameworks

**DOI:** 10.1038/s41746-026-02784-2

**Published:** 2026-05-28

**Authors:** Dipendra Pant, Thomas Brox Røst, Harald Krüger, Pieter Jelle Toussaint, Øystein Nytrø

**Affiliations:** 1 Department of Computer Science, Norwegian University of Science and Technology, Trondheim, Norway; 2 Department of Child and Adolescent Psychiatry, Clinic of Mental Health Care, St. Olav University Hospital, Trondheim, Norway; 3 Legal Division, Sykehusinnkjøp HF, Vadsø, Norway; 4 Present Address: Department of Computer Science, UiT The Arctic University of Norway, Tromsø, Norway

**Keywords:** Health care, Scientific community

## Abstract

Secondary use of health records is vital for research, quality improvement, innovation, but it must comply with complex legal, ethical, and security requirements. In Norway and the European Economic Area (EEA), this involves navigating national health legislation alongside the European Union (EU) regulations. For secondary use in Norway, we identified sixteen regulatory documents, notably the European Health Data Space (EHDS) regulation. We synthesized these documents into a nine-step guideline with checklists to operationalize secondary use as a structured workflow, encompassing Secure Processing Environments (SPE), security controls, lawful consent, ethical review, contractual, technical safeguards, and auditability. Additionally, we provide recommendations for applying the guideline in other EEA countries. The guideline reflects on the phased application of the EHDS, SPE requirements, and the Artificial Intelligence (AI) act. As a practice-oriented synthesis, it offers a practical starting point for navigating lawful secondary use of health records.

## Introduction

Within the European Union (EU) and the European Economic Area (EEA), enabling secondary use of health records for improving healthcare research and innovation is an actively pursued goal^1,2^. A key consideration for achieving this is to ensure that regulatory oversight and ethical safeguards are in place^3,4^. Many EU/EEA countries are known for universal, publicly funded healthcare^5,6^. Regulations play a vital role in safeguarding basic human rights, such as access to healthcare, the right to privacy and data protection, but the EU/EEA encompasses many countries across a large geographical area which complicates consistent application. The European Health Data Space (EHDS) regulation is an EU and EEA-relevant regulation. It states secondary use involves using health records for purposes beyond the original intent of providing primary care (EHDS Chapter IV Arts. 51, 53)^3,7^. The potential for secondary use of health records for research, public health, innovation, Artificial Intelligence (AI) product and service development, policy-making, and other purposes is recognized at the national^8^ and international levels^1,3^. However, its application faces significant legal, information security, and computing infrastructure challenges, and achieving secondary use in practice is not straightforward^4,9^. The EU/EEA member states maintain extensive Electronic Health Records (EHR), with twenty EU member states (74%) reporting that 80–100% of their population can access their health records through the provided access services^2^. Yet secondary use remains underutilized^1,10^. Improvements in algorithms, tools, and technologies, advances in interoperability^11,12^, system development^13,14^, and security^15^ will make it easier to automatically navigate and interpret health records. This, together with the EU’s efforts under the EHDS to harmonize health record systems across member states, is expected to support fragmented healthcare infrastructures by enabling seamless and secure cross-border access and secondary use of health records across the EU.

The EU’s efforts through EHDS highlight how common regulatory and technical frameworks can mitigate fragmentation in nationally organized specialist healthcare systems. For instance, in Norway, specialist healthcare is structured into four regional health authorities, each operating distinct EHR systems, contributing to a fragmented information infrastructure. In the Norwegian healthcare sector, there is a bewildering landscape of national and EU legislation, norms, standards, and recommendations that must be adhered to varying degrees. EU legislation is legally binding in Norway under the EEA agreement. This landscape is further complicated by inconsistent interpretations among data controllers^16^, evolving policy, and ambiguous regulatory reforms. For example, a Norwegian regulation^17^ mentions the use of a “closed and secure analysis infrastructure”, yet the official guide^18^ offers no further definition or specification. Another example is Norway’s attempt to introduce a centralized Health Analysis Platform (HAP), which was put on hold^19^. A reason is the implications of the EU’s ‘Schrems II’ decision, which created legal uncertainty around using cloud services involving transfers of personal data to third countries. Instead, Norway strengthened the national health data service *(i.e., Helsedataservice)* as a unified access point for health data, while the idea of a fully centralized national analytics platform was put on pause^19,20^. Such shifts create uncertainty in infrastructure planning and leave operational specifications and roles open to interpretation.

This study provides guideline, including steps, checklists, and flowcharts, and EU/EEA transferability recommendations to navigate the lawful, safe, and ethical secondary use of health records in Norway. Unless explicitly mentioned, all references to legislation, regulatory, and jurisdictional context in this study pertain specifically to Norway. The guideline are intended to align with current Norwegian regulations and EU-level international laws and regulations. Although the guideline are grounded in Norwegian and EU legislation and norms, they shall be transferable to other comparable jurisdictions, such as EU/EEA countries, with adaptations. The following paragraphs define the key terms health records, secondary use, EHDS, and outline the scope of the study.

**Health records** encompass diverse information about an individual’s health, provided or planned care, and related services, including electronic health data, EHRs, and patient journals. Electronic health data gathered in EHRs include a person’s medical history, diagnoses, treatments, medications, allergies, vaccinations, radiology images, laboratory results, and other medical data, which are often distributed across different healthcare actors such as general practitioners, hospitals, pharmacies, and care services (EHDS Recital 7)^21^. The EHR is the technical information system used to create and store the patient journal. The patient journal is a record of an individual’s health history, which consists of assessments, care, and interventions provided to a patient by separate health service providers, and they are legally mandated records that are processed when providing healthcare^22^. Under EU’s General Data Protection Regulation (GDPR) Recital 51 and Article 9(1), health records are sensitive personal data, which requires higher protection and must be processed in a manner that protects patient confidentiality and integrity (Patient Records Act (PRA) §15)^23^. In Norway, GDPR is implemented by the Personal Data Act (PDA)^24^.

**Secondary use** refers to the use of health records beyond the original collection purpose, such as research, quality improvement, and policy planning^3,7,25^. The primary use of health records is for direct patient care. The **EHDS** regulation establishes the legal framework for secondary use of health records within the EU/EEA, including rules governing access authorization and secure data processing^3^. It establishes a harmonized framework for secondary use involving Health Data Access Bodies (HDABs) and Secure Processing Environments (SPEs). Under the EHDS, an HDAB is the designated national authority responsible for receiving and assessing secondary-use applications and issuing data access permits (EHDS Arts. 55-57). An SPE is a controlled technical environment in which approved secondary-use data processing must take place under defined technical and organizational safeguards (EHDS Art. 73). Together, these are intended to enable lawful access to health records while ensuring privacy, security, and accountability in secondary-use processing; see the EHDS and data governance subsection below for further details.

The **scope** of this guideline is to assist anyone planning secondary use of health records. This includes clinicians, researchers, data stewards, Information Technology (IT) and security staff, administrators, data scientists, analysts, AI/Machine Learning (ML) engineers, and software developers. The guideline is also intended as a shared reference for multidisciplinary teams, institutional decision-makers, controllers, data holders, HDABs, SPE operators, and information-security, ethics, and data-protection authorities. The guideline is designed for project scoping, governance coordination, and early regulatory orientation, rather than as a substitute for case-specific legal or ethical advice. Guided by EHDS Chapter IV (Art. 53)^3,7^ the guideline focuses on the secondary use for: (i) Research: generating new knowledge using scientific methods, (ii) Innovation/AI development: developing or training a new tool, product, service, algorithm, or AI model, (iii) Quality improvement: monitoring, evaluating, or improving the quality of a specific health service within own institution, (iv) Statistics/Policy planning: health and care service administration, planning, management, or statistics at a local, regional, or national level. In projects combining research, innovation, quality improvement, and/or statistics/policy planning, the same guideline apply. This guideline can be applied to all kinds of information in health records, except when data is not fully embedded in EHR. However, in circumstances where the processing of EHR-embedded data involves specific data categories such as biological materials, specialized regulatory regimes such as the Biotechnology^26^ and Biobank Act^27,28^, may be overlapping, complementary or even conflicting with the guideline.

## Results

### EHDS and data governance

**Fig. 1:**
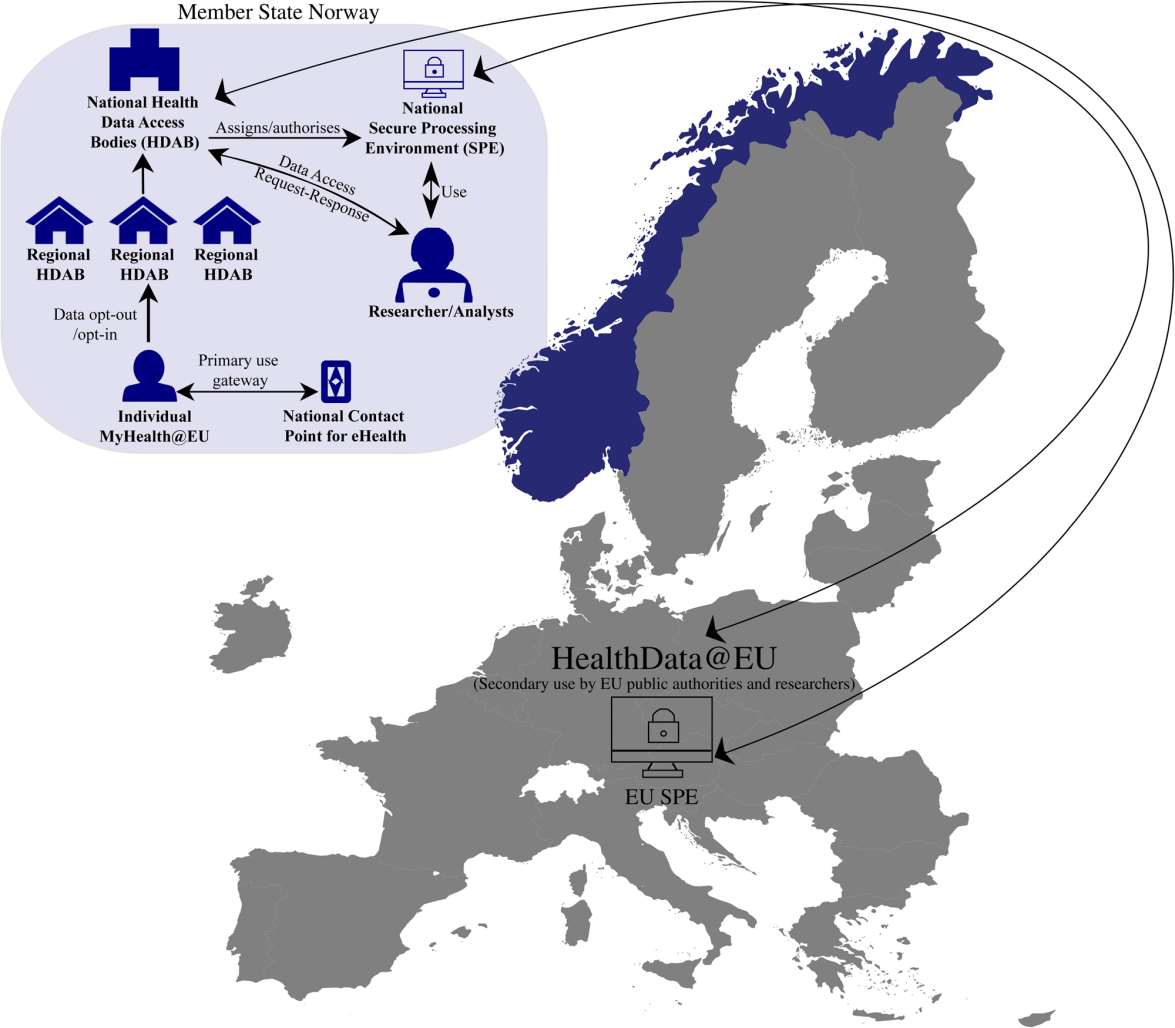
EHDS infrastructure for secondary use between Norway and the EU. Norway, as a member state with a national data access body and a SPE, uses and interacts with the EU-level HealthData@EU and EU SPE infrastructure to perform secondary use. Map drawn using Geographic Information System of the European Commission (2024), CNTR_RG_10M_2024_4326.geojson.

The EHDS governance shown in Fig. 1 informed the guideline’s design by clarifying how responsibilities are distributed among data holders, HDABs, and SPE operators and by identifying regulatory decision points within the secondary-use workflow. These governance characteristics directly shaped the guideline design, particularly the separation of roles related to ethical approval, data access authorization, and secure data processing. This separation is reflected across guideline steps and operationalized through the RACI matrix (Table 1).

**Table 1:** Roles and responsibilities according to the Responsible, Accountable, Consulted, and Informed (RACI) matrix, including primary responsibility per guideline step aligned with the EHDS

Task	Controller	Processor	PI	Analyst	Ethics Committee	Registry controller	Data steward	InfoSec	DPO	HDS / HAP	Patient	Primary responsibility (EHDS)
Project classification	A/R	I	R	I	C	I	C	C	C	I	I/C	Secondary data user (PI / applicant)
Legal basis	A/R	I	C/R	I	C	I	C	I	C	I	C	Data holder / controller
Ethics approval	I	I	R	I	A	I	I	I	C	I	C	Secondary data user (ethics applicant)
Data access application	C	I	R	I	C	A/R	R	C	C	R/C	I	Data holder / controller (with HDAB permit)
Data security and privacy	A	C	I	I	I	I	C	R	C	A/R	I	HDAB / SPE operator
Compliance monitoring and auditing	A/R	C	I	I	C	C	C	R	C	A/R	C	HDAB (oversight) / controller
Analysis and AI development	C	I	A/R	R	C	I	C	I	I	I	I/C	Secondary data user (inside SPE)
Data minimization and quality	A/R	I	R	R	I	C	R	C	C	I	I	Shared: controller + data user
Dissemination	A/R	I	R	R	C/A	I	R	C	C	I	I	Secondary data user (under permit conditions)
Close-out, retention, and deletion	A/R	R	R	R	I	C	R	C	C	A/R	I	Secondary data user (under permit conditions)
Transparency & communication (public or data subjects)	A	I	C/R	I	C	I	R	I	C	I	I	Shared: data controller (legal duty), HDAB (system-level transparency)
Incident & breach management	A	C	I	I	I	I	I	R	C	A/R	I	Shared: data controller (accountable), SPE/processor (operational)

*R* Responsible, *A* Accountable, *C* Consulted, *I* Informed.

Governance-wise, Fig. 1 shows two complementary components of EHDS and the overall framework for secondary use between Norway and the EU states. The first component, ‘MyHealth@EU’ supports cross-border exchange in direct care (primary care). The second component, ‘HealthData@EU’ enables secondary use via national HDAB and SPE (EHDS Chapter IV)^3^. Within the EHDS governance framework, HDABs represent the central regulatory decision point for secondary use, linking ethical review, data permit issuance, and controlled access mechanisms. HDAB ensures that data processing takes place in an accredited SPE once a secondary-use request is approved. The HDAB does not supersede national ethics committees; ethical review remains national. Data permits are therefore issued only after any ethically required approvals have been obtained, ensuring complementary but clearly separated responsibilities. HDABs may further interact with trusted data holders or controllers and EU-level governance entities as part of the secondary-use authorization process. Under the EHDS, HDABs also maintain national metadata catalogues describing available health datasets, their characteristics, access conditions, and quality attributes (EHDS Arts. 77, 79). These catalogues support discoverability and transparency and are a prerequisite for efficient secondary use. For the secure health records access with accountability, and to use EHDS data governance (comprising of ‘MyHealth@EU’, ‘HealthData@EU’, National HDAB, Regional/Local HDAB, data owner, and researcher/analysts), the different entities requires policies for access, storage, ownership, and sharing; identifying who does secondary use, who pays, and who gets benefits from secondary use.

Within the EHDS governance framework, the requirement that all approved secondary-use processing occurs within accredited SPE establishes a core accountability mechanism. Inside such environments, users operate under strict technical and organizational controls and may export only non-personal, anonymized outputs in accordance with the conditions defined in the data permit. SPE operators must implement safeguards including role-based access control, logging, monitoring, and export control mechanisms. Together, these measures enable privacy-preserving and auditable reuse of health records^3^.

Norway is already headed in the direction of developing and utilizing SPEs for sensitive health data and aligning policies for access, storage, sharing/ownership, accountability, and clarifying who may conduct secondary use, who pays, who benefits, and major entities involved^29^.

**Data controller** status, or ownership of the health record and the maintained EHR system (including its secondary uses), typically rests with the health-care institution, such as a public health trust. According to the Norwegian PRA (PRA §2e), the controller has ultimate legal responsibility for the health records it collects, ensuring compliance with data protection regulations, while the patient or data subject retains statutory rights to access, correct, and manage their health records^30^. Secondary use under EHDS primarily requires (i) Data holders or controllers, they determine the purposes and means for processing health records, and the access is provided via the HDAB. (ii) The SPE, which has not yet been implemented, and its obligation applies at a later date. We refer to the currently used sensitive data analysis platforms in Norway as the Secure Analysis Environment (SAE). In Norway, ‘HUNT Cloud’ by the Norwegian University of Science and Technology, Services for Sensitive Data (TSD) by the University of Oslo, and Secure Access to Research Data and E-infrastructure (SAFE) by the University of Bergen are used as SAEs^29^. These SAE can be considered a specific lab or enclave within the SPE infrastructure where scientific work is performed, but SPE remains the EHDS legal term^29^. And maybe the SAE can be upgraded to SPE upon fulfilling the technical specifications. Towards European Health Data Space-2 (TEHDAS2)^31^ is an example of a SPE project at the EU-level. In general GDPR mentions (iii) Data processor, an entity or service provider that processes personal data only on behalf of the data controller and according to its instructions, this includes external cloud platforms, EHR system vendors, IT or cloud service providers, data integration services, and researchers or research platforms used to process data under the controller’s mandate with a data processing agreement as per GDPR article 28.

**Who performs secondary use** typically includes authorized researchers and clinicians involved in innovation, research, or quality improvement, provided they have obtained HDAB data access, ethical clearance, and have formal data processing agreements in place AI and data teams for model development, validation, and quality improvement within SPE using de-identified or pseudonymized data, and identifiable data only when legally permitted, with strict minimization and no re-identification.

**Benefactors and benefits** are primarily driven by funding from research grants, government bodies, or university collaborations, and the resulting outcomes should bring profit and advantages to the (i) Patients and society through improved health outcomes and policy insights; (ii) the healthcare system via optimized treatment pathways and resource allocation; (iii) Researchers through publications and intellectual property; and (iv) the institution through gains in expertise and reputation, with any commercial revenues remaining subject to public research guidelines.

**Roles** and responsibilities for the secondary use of health records are defined in Table 1, which are formal or institutional roles and responsibilities. The Table 1 outlines the separation of responsibilities, accountability, and consultation required, as well as the entities to be informed and the primary responsibility holders under EHDS at each step. Some roles, such as controller, processor, and ethical approval, have been discussed above and are mandated by regulations, while others are institution or project-specific. Some roles not mentioned in the above sections are (i) Data Protection Officer (DPO), a formally mandated independent advisory/monitoring role per GDPR; they review Data Protection Impact Assessment (DPIA) and incident response; coordinate with the data protection authority where needed (GDPR Arts. 35, 37,38, 39). (ii) The Principal Investigator (PI) and/or Project Manager (PM) manages the research protocol, ensures ethical approvals, and coordinates with legal and ethical bodies. An analyst collaborates with the PI/PM to assist with data cleaning, standardization, analysis, and modeling. The clinical liaison provides domain expertise and ensures the data context is adequately understood by analysts. (iii) A data steward ensures data quality, manages metadata, and facilitates transparency and interoperability across systems. (iv) An Information Security (InfoSec) officer is a strategic executive typically consulted to set security strategy, ensure the confidentiality, integrity, availability of health records, oversee risk management, compliance, and respond to incidents. They align security with organizational objectives and patient safety. (v) The Health Data Service (HDS) functions as an access provider, similar to the HDAB in the EHDS. The Health Analysis Platform (HAP) corresponds to the SPE/SAE in the EHDS and operates on behalf of the HDAB.

### Guideline

**Fig. 2:**
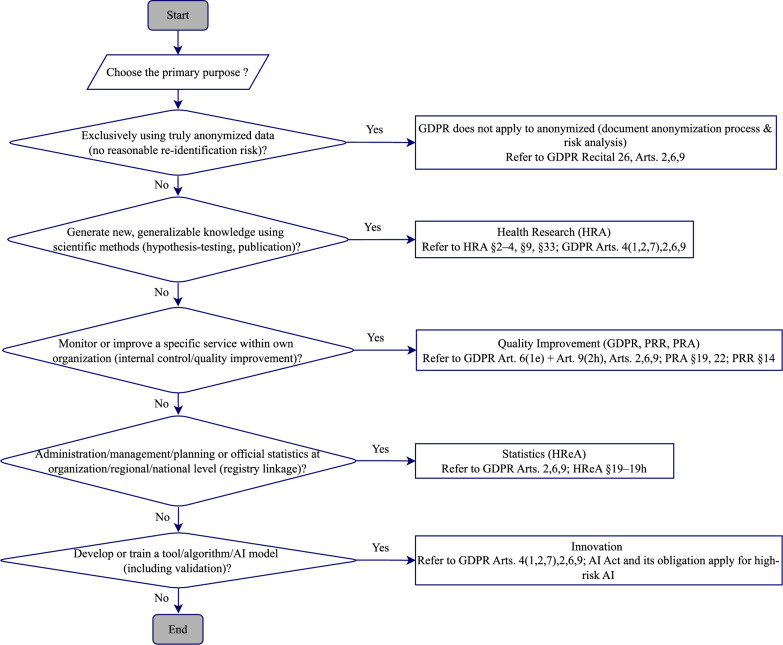
Decision path to identify the purpose of secondary use. Diamonds represent decision points, and rectangles represent primary purposes.

**Fig. 3:**
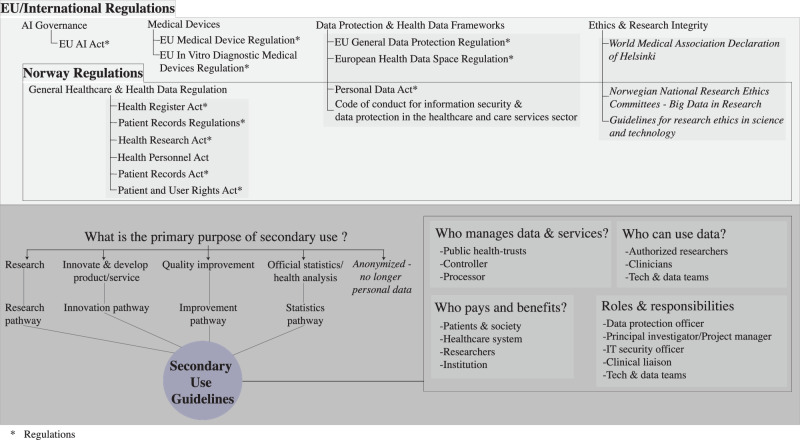
Laws, regulations, and the focus shaping the guideline. The light gray area shows the EU and Norway-level regulations, while dark gray shows the aspects that shaped the guideline, with a focus on the primary purpose, data governance, roles, benefits, and responsibilities.

The primary purposes of secondary use of health records are outlined in Fig. 2 in accordance with Fig. 3. Anonymized data may be accessed via HDABs. However, the GDPR doesn’t apply to data that has been irreversibly anonymized. Mainly EHDS, GDPR at the EU level and other regulations in regulatory and legal context section supplement secondary use for research, obtaining ethical approval, consent or dispensation, and adhering to the Helsinki Declaration before data access. For quality improvement, the GDPR, national regulations on health records, and patient and user rights are generally applicable (e.g., Patient Records Regulations (PRR), PRA). When the purpose is public administration and statistics, the health registry regulations (e.g., Health Register Act (HReA)) regulate the availability and use of registry data. For the innovation pathway focused on product and service development, the EU AI Act, and Medical Device Regulation (MDR)/In Vitro Diagnostic Medical Device Regulation (IVDR) for medical devices or software as medical devices can apply. Also, research leading to innovation and AI system development may fall within the scope of the national research acts (e.g., Health Research Act (HRA)).

**Fig. 4:**
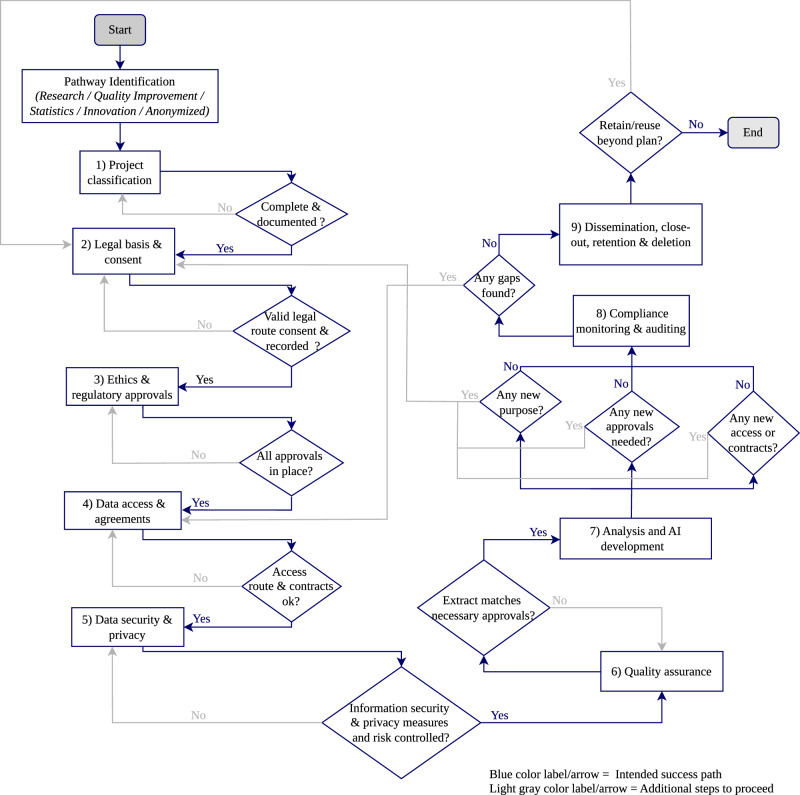
Navigating secondary use of health records in Norway, with potential transferability to the EEA. Each box represents a step in the guideline, while diamonds represent conditional statements. Pathway identification is shown in Fig. 2. The dark blue lines indicate the intended path, ensuring that all steps in the guideline are followed. Light gray lines indicate that additional steps are needed before proceeding.

Fig. 4 guides the flow of the guideline from steps 1 to 9 upon pathway identification through Fig. 2. Steps 1-6, 8, and 9 apply across all EHDS secondary-use purposes, including research, quality improvement, statistics, and policy planning, whereas step 7 applies when secondary use involves algorithmic analysis or development of AI systems. A checklist of recommended tasks for the structured, lawful secondary use of health records is provided for each step. The checklist contains clickable references. Because each step may include tasks performed by different actors, checklist items should be read in conjunction with Table 1.

#### Guideline for secondary use of health records in Norway, potential transferability to EEA

Step1 Project classification: Classify the project and record the controller(s), processor(s), purpose, dataset(s), identifiability.

□ Classify per Fig. 2, note the purpose, identify controller(s), processor(s), set roles. Refer to HRA §2-scope, §4-definitions, GDPR Art. 4-classification, 4(7)-controller, Table 1 and note EHDS Art. 53-permitted purposes.

□ Identify the data status to differentiate into personal data, special category-health/genetic/biometric, or truly anonymized. Note that pseudonymized data remains personal data, and genetics falls under biotechnology. Refer to GDPR Art. 9(1)-special category, 4(5)-pseudonymized, GDPR Recital 26-anonymized, HReA §2f-pseudonymized,Biotechnology Act.

□ If medical/health research pathway the health research act applies (HRA §2, 4) and ethics committee (e.g., Regional committees for medical and health research ethics (i.e., REK)) decides the scope.

□ If using health registers, record relevant legal registry restrictions per HReA §8-11.

□ For non-medical/health research projects, consult health trusts, follow Fig. 4, consult the DPO, and retain a written rationale. Refer to GDPR Art. 5(2), 24(1).

□ Plan periodic re-review of classification as technology and law changes, comply with the accountability principle (GDPR Art. 5(2)).

Step 2 Legal basis and consent: Determine the legal basis, address confidentiality requirements, including any applicable exemptions or consent; document the decisions.

□ Public bodies may perform secondary use for tasks either in the public interest or with official authority (GDPR Art. 6(1e)) or for research/statistics (GDPR Art. 9(2j)) with safeguards (GDPR Art. 89(1)) or healthcare/management/quality improvement (GDPR Art. 9(2h)). Provided that an appropriate supplementary legal basis under GDPR Art. 9(2) and national law (e.g., Health Personnel Act (HPA), PRA, or other sector-specific legislation) is present.

□ Complete a DPIA where processing is likely to result in a high-risk project (GDPR Art. 35).

□ Ensure confidentiality under health law, either valid consent, or dispensation from consent and confidentiality, the dispensation may apply if the use is for archival, research, or statistics in the public interest, and societal benefits outweigh individual risk. Refer to HPA §21-duty of confidentiality, HPA §29 & HReA §19e-dispensation.

□ If using consent, ensure GDPR or HRA valid consent with all required information signed by all participants, and that informing about secondary use is covered (or re-consent where required and withdraw consent). Refer to GDPR Arts. 7,13-14-information duties, HRA §13-consent.

Step 3 Ethics and regulatory approval: Submit relevant research protocol, consent/waiver justification, data-protection plan, risk assessment, privacy safeguards. And obtain the required approvals before accessing the data.

□ Ensure ethical approval (e.g., REK) is obtained or exempted before access/use of health records for research (HRA §9, §33)?

□ Ensure recommendation from ethics committee and regulators (e.g., REK), data protection authority (i.e., Datatilsynet), registry/data holders (e.g., linkage constraints) are taken into account.

□ Have Record of Processing Activities (RoPA) & transparency notices ready to comply with data protection regulations. Refer to GDPR Arts. 30-RoPA, 13-14(transparency).

Step 4 Data access and agreements: Obtain a Data Sharing Agreement/Data User Agreement (DUA/DSA) or equivalent with the data provider; specify scope, purpose, duration, security and confidentiality clause, including post-hoc control, return or secure post-project destruction of data. Secure the right access pathway and contracts before transfer/use.

□ Ensure data controller and processor have technical, organizational information security, including access control (GDPR Art. 32, PRA §22, Code of conduct for information security and data protection (NORMEN) section 5.2, 5.4.4) & logging with access lists and access logs maintained (PRR §14).

□ Ensure all team members signed, handling data with confidentiality agreements and/or completed training on duty of confidentiality (HPA §21).

□ Use SPE/SAE for analysis and development (e.g., TSD, HUNT Cloud, SAFE in Norway^18^).

□ Ensure that transfer of health records to the SPE or SAE is performed by the controller or data holder through approved secure channels, aligning with security of processing (GDPR Art. 32), *NORMEN* (logging, access control, encryption appropriate to risk and sector).

□ If data comes from an external source, such as outside your institution, like national health registries/health trusts.

– Obtain data access agreements, dispensation (HReA §19e / HPA §29).

– Have data processing agreements, data sharing/user agreement specifying purpose, scope, duration, security, and destruction/return signed (GDPR Arts. 28(3)-processor contract, 26- joint controllers).

□ Determine the maximum permitted period for holding and accessing the data, ensure compliance with the approved data retention period and access window (HReA §19f).

□ Confirm that the data agreement aligns with the secure processing environment checklists^18^.

□ Consult the HDAB data catalogue (or where available the foreseen EU dataset catalogue), including dataset descriptions, to identify relevant data sources, assess availability, data characteristics, and applicable access conditions prior to submitting a data-access application (EHDS Arts. 77, 79).

□ Note EHDS alignments (for cross-border and secondary use), HDAB duties (EHDS Arts. 55 - 59); plan for data permit (EHDS Arts. 66-68) and use of a SPE (EHDS Arts. 73,75).

Step 5 Data security and privacy: Implement defense in depth, ensure privacy protection, and document controls.

□ Pseudonymize where possible by removing or replacing direct identifiers, and keep keys separate (GDPR Art. 4(5)-pseudonymization).

□ Encrypted storage (at rest and in transit, no protected health information on unencrypted devices) and safeguarded servers (role-based access control, multi-factor authentication, network segregation, key management), ensure information security controls, logging practice and risk-based log review. Refer to GDPR Art. 32, PRA S22, PRR §14, NORMEN factsheets, and *section 3.2, 5.3.5, 5.2.1, 5.4.4*^32^.

□ Unless anonymized, derived datasets with personal data get the same data protection and security as the original (GDPR Art. 5(1f), 32).

□ Ensure DPIA is conducted before data access or analysis (e.g., for large-scale health records, innovative AI, vulnerable groups such as childrens) or documented rationale if not (GDPR Art. 35).

□ DPO consultation is recommended where required; record advice, and implement recommendations if any (GDPR Arts. 37-39).

□ If sharing a dataset within Norway or the EU, verify permission, & GDPR lawful basis and core principles, respect intellectual property, licensing, and ethics committee terms. Refer to GDPR Art. 5(1b-c), GDPR Art. 6(1-legal basis), GDPR Arts. 44-49, HRA §33-conditions.

□ If transferring personal data outside the EEA, verify permission, confirm lawful basis (GDPR Arts. 6, 9), use an allowed mechanism (GDPR Arts. 44-46 on adequacy, GDPR Art. 46 on Standard contractual clauses (SCCs) safeguards, GDPR Art. 49 on derogations), assess level of protection, laws in the new country, the infrastructure, processor, subcontractors involved and document a data transfer impact assessment with management and mitigation strategy. Keep necessary approvals and maintain logs and documentation. Refer to EHDS Arts. 88 -90, data protection authority (i.e, Datatilsynet).

□ If third-party tools/vendors are involved, sign data processor agreements and vet safeguards (GDPR Art. 28).

□ In case of data breach, notify the data protection authority within 72 hours (GDPR Art. 33) where required, and assess the duty to inform data subjects (GDPR Art. 34).

□ Periodically review security measures (including SPE/SAE) to incorporate new threats or updates in technology and regulation.

□ Note that for many healthcare providers, EU Network and Information Systems Directive-2 (NIS 2) directives impose cybersecurity and incident reporting obligations, which shall be integrated into SPE governance.

□ Note when secondary use of data, supports MDR/IVDR-regulated software as a medical device, ensure that security controls and monitoring also fulfil MDR/IVDR post-market surveillance and AI act requirements for high-risk AI systems.

Step 6 Data minimization and quality: Keep to what’s necessary and verify extracts.

□ Identify and extract only the data fields necessary or approved. Best practice is to avoid excessive and unrelated personal data collection in original and derived datasets, from a computational, security, and compliance perspective. Apply data minimization and purpose limitation as per agreed purpose (GDPR Art. 5(1b-c)).

□ Perform post-extraction check and sweeps for unexpected/disallowed content (e.g., check free text, images) unless approved (GDPR Art. 5(1c), 25).

□ Conform to approvals, scope and project objectives. Set periodic data quality review aligned with emerging standards/regulatory updates (GDPR Art. 5(1d, 2)).

Step 7 Analysis and AI development: Analyse data strictly within SPE/SAE; forbid re-identification unless authorized.

□ Use only validated analytical tools and methods that comply with the approved protocol of a SPE/SAE do not attempt re-identification unless legally authorized (GDPR Art. 5(1a-b)).

□ Maintain a comprehensive preprocessing documentation (on any cleaning/transformation/filtering/anonymity applied) for transparency and reproducibility (AI Act Art. 10, GDPR Art. 5(2)), and technical documentation (AI Act Arts. 53(1a-d)), AI Act Annex XI-XII.

□ Perform an AI risk classification assessment, and document assessment (AI Act Art. 6-7(classification framework), Annex III-categories). For high-risk AI,

– Implement controls, maintain technology documentation, define human oversight (AI Act Art. 9-14); verify and validate performance, model drift, cybersecurity levels (AI Act Art. 15).

– Apply data governance for training/validation/testing to ensure data is representative, relevant, accurate, and bias-assessed. Refer to AI Act Art. 10(2); GDPR Art. 5(1d-accuracy).

– Include human-in-the-loop reviews with a clear human oversight and manual intervention defined, such as how clinicians/staff supervise and can override, intervene AI output and decisions (AI Act Art. 14).

□ For non-high-risk AI, adopt good practice on data quality (provenance, representativeness, documentation), bias (subgroup evaluation, mitigation, monitoring), and transparency (clear AI use notice, limitations, human oversight), consider voluntary compliance, and ethical guidelines. Refer to GDPR Art. 5(1a-d), AI Act Arts. 95 - 96 (voluntary codes of conduct).

□ Maintain the technical documentation and record keeping of the AI system, including model description, algorithms, training data source, and intended purpose, evaluate and validate the model. Refer to AI Act Art. 11-tech documentation, Art. 12-record keeping.

□ For AI system maintain obligations on risk management (AI Act Art. 9), data & data governance (Art. 10), tech documentation (Art. 11), record-keeping (Art. 12), transparency (Art. 13), human oversight (Art. 14), accuracy/cybersecurity (Art. 15). It is recommended to plan now, but it applies when placing on the EU market/putting into service.

□ If developing an AI model, verify permission & GDPR lawful basis and core principles (AI Act Art. 2(7)), remove personal data or ensure model is vetted and validated, respect intellectual property, licensing and ethics committee terms. Refer to GDPR Art. 5(1b-c), GDPR Art. 6(1-legal basis), GDPR Arts. 44-49.

□ If deploying the AI system on EU, are all compliance met and documents in place (conformity assessment or European Conformity(CE) marking, for high risk AI registration in the EU AI database where required. Refer to AI Act Arts. 30 & 43 (conformity assessment/market), medical device regulation/in vitro diagnostic regulation, where applicable; see EU MDR 2017/745 and the MDR software guidance "Qualification and classification of software”).

□ Implement and document risk management, bias mitigation in data interpretation and reflect upon the analyzed results. Refer to AI Act Art. 9-risk management, Art. 10-bias(2e, f, 5); GDPR Art. 35 (DPIA).

□ Excluding data exhibiting systematic malpractice from clinical settings and running fairness checks (e.g., demographic parity, equalized odds, or subgroup analysis), performing regular bias audits and compliance reviews. Refer to bias audits and compliance reviews (AI Act Arts. 9, 10(2e), 15), data subject rights if AI model decisions impact individuals (GDPR Arts. 12-22).

□ Have a process for periodic reassessment and review of the AI systems, risk classification as regulations and technology evolve.

Step 8 Compliance monitoring and auditing:

□ Have an internal audit (by the external institutions or health trust) and remediate any discrepancies and compliance issues. Refer to GDPR Art. 5(2-accountability), Art. 24-controller’s responsibility.

□ Maintain a compliance report with all project documents, approvals, permits, and agreements.

□ Have all data extraction from source systems been logged as required, this includes ensuring that any query or export from EHR or registry systems is recorded, including who performed it and when.

□ Perform log extraction access to source system, and periodic log review (PRA §22, PRR §14, NORMEN 5.4.4), it is recommended to set a risk-based review cadence in policy.

□ Ensure data is used only for the approved protocol and seek amendments before use for other purposes or new analyses. Refer to GDPR Art. 5(1b-purpose limitation), HRA §33 (conditions).

□ Review and update the audit and compliance process periodically, aligning with changes in legal and technological aspects.

Step 9 Dissemination, close-out, retention, and deletion: Disseminate only safe anonymized outputs, close formally, and implement secure archival, data retention, and deletion.

□ Before releasing results (internally or externally), checked that no individual can be identified from the disseminated output (GDPR Art. 5(1c), Art. 89(1)).

□ Have publication include required statements about ethics & data-protection statements (e.g., -"Approved by REK, reference number”, “Data processed in compliance with GDPR”, for clinical research Helsinki ethical statements).

□ If an AI product or service discloses intended use, limitations, validation status, transparency (AI Act Art. 13).

□ Fulfill any reporting obligations, like for research, submit required progress/final reports to authorities, registry, or funders. For AI deployments, notify the EU AI database register if required (AI Act Chapter III).

□ Note individuals’ rights under the EHDS, including the right to opt-out of secondary use (EHDS Art. 71) and the right of access, correction, and portability of electronic health data (EHDS Arts. 3 -9) are operationalized and documented.

□ For the post-project closure:

– Ensure all personal data are deleted irreversibly or anonymized at the end of the retention period. Best practice is to double-check backups, personal laptops, or secondary copies to ensure no stray data remains. Document the date and method of deletion/anonymization for accountability. Refer to GDPR Art. 5(1e-storage limitation), Art. 5(2-accountability).

– Archive all key documentation securely (approvals, consent, analysis scripts, final reports, DPIA) as per institutional policy, often 5-10 years without identifiable raw data (GDPR Art. 5(1e), Art. 89(1)).

– If retaining/reusing data, record the new legal basis (e.g., renewed consent, extended ethics approval, or transfer into a lawful data bank/registry). Refer to GDPR Art. 6(1), Art. 9(2-processing); HRA §13-14, 33 (consent) and health registry rules (i.e., Helseregisterloven).

– Conduct a final debrief meeting. Make a note of any best practices learned or recommendations.

– If applicable, notify relevant bodies that the project is complete, such as informing data protection authority, ethics committee that the study is concluded and data is destroyed, update the institution’s record of processing to mark the project as finished.

– If an AI model continues in clinical use, govern it under operational protocols (monitoring, maintenance, fallback), and ensure required product/AI act compliance. If discontinued, ensure no personal data persists via model artifacts (AI Act Arts. 9-15, 30 & 43 (requirements & conformity)).

To support transferability of the guideline to EU/EEA, Table 2 contains recommendations, mapping the guideline to one of three categories: (i) EU/EEA regulatory considerations, (ii) Adaptations within EU/EEA, (iii) Norway-specific examples. We emphasize that while the guideline can be broadly transferable, concrete application will differ across member states due to national law, governance arrangements, and infrastructure. As an illustrative example outside Norway, a university medical center in the Netherlands is planning a retrospective EHR study. They should first map out the responsible ones for ethics review, access authorization, SPEs, and compliance oversight at the national level, and then apply the guideline. In the Netherlands, ethics committees may include the Central Committee on Research Involving Human Subjects (CCMO) or the Medical Research Ethics Committees (MRECs). Data access would initially be handled through the hospital controller and relevant data holders, while analysis could be performed within the SPE/SAE, such as the Netherlands’ Central Bureau of Statistics (CBS) microdata environment or Secure ANalysis Environment (SANE). EHDS implementation, including the Netherlands’ health data access body (HDAB-NL) and a work package on SPEs^33^, is still ongoing in the Netherlands. Once EHDS implementation matures in the Netherlands, HDAB-NL is expected to provide the national access body layer for this illustrative example.

**Table 2:** Secondary use guidelines EU/EEA transferability recommendations

Core question	EU/EEA regulatory considerations	How to adapt in other EU/EEA countries?	Norway-specific
Are the data and roles in scope?	GDPR defines personal data and special categories; EHDS defines health data holders, data users, secondary use purposes, and SPEs; and national ethics and healthcare regulations designate roles (e.g., ethics committees and supervisory authorities).	Identify health records and main public controllers; list national ethics bodies and supervisory authorities; identify SPE available for secondary use.	Health records or sensitive personal data; roles include health trusts as controllers and processors, ethics committees, the National HDAB, national registries, DPOs, and Norwegian SAEs (e.g., TSD, HUNT Cloud, and SAFE).
Is the task for research, quality improvement, statistics, or innovation?	EHDS purpose-based categories for secondary use (e.g., research, public health, policymaking, and innovation); GDPR supports research, statistics, and public-interest purposes with safeguards (e.g., using an identifiable health record to produce knowledge would be research and require safeguards).	Map each EHDS purpose category to national research, registry law, and quality-improvement frameworks, ask HDAB; check ethics committee review and internal quality procedures where required.	Classification guided by HRA (i.e., health research), HReA (i.e., registries and statistics), PRA/PRR (i.e., care and quality improvement), and national interpretations of “quality improvement” vs “research” (e.g., REK approved study analyzing patient outcomes can fall under the HRA).
What is the legal basis for processing, and is patient consent required?	GDPR (e.g., research or public-interest derogations); EHDS permitted purpose for secondary use; the Helsinki Declaration sets ethics standards.	Identify national health research acts, registry laws, or sectoral laws; consult national adoption of GDPR for research consent, broad consent, opt-out, or a statutory basis applies for secondary use.	HRA and PDA defines research consent and derogations; the PRA, Patient and User Rights Act (PURA), HReA, and PDA specify lawful basis and confidentiality rules.
Does the task need ethics approval or other regulatory sign-off?	Ethics committee review for health and medical research is required under national law and the Helsinki Declaration; the data protection authorities and DPO may be consulted for high-risk processing (e.g., DPIA).	Map national ethics structures; specify when ethics committee review is mandatory or optional for registry, quality improvement, limited or low risk AI-development.	REK serves as the ethics committee for health research, and its decisions determine whether HRA applies to health record-based projects, the National Research Ethics Committees (FEK) handles appeals.
What is the data access channel and agreements?	Access permit through HDAB and processing in SPE; data sharing agreements must follow GDPR and EHDS	Identify national HDABs and SPEs; specify how controllers route requests through HDABs and which templates or permits govern EHR and registry access.	The Norwegian Health Data Service evolving into the Norwegian HDAB; secure access via SPEs; HReA, PRA, and PRR add conditions for registries and EHRs.
What minimum technical and organizational measures are required?	GDPR and NIS 2 obligations; EHDS SPE requirements; AI act and MDR/IVDR impose life cycle security requirements for high-risk AI.	Map EU obligations to national cybersecurity and e-health standards; identify national baseline controls for SPEs.	*NORMEN* provides Norwegian healthcare sector information security, secure infrastructures, and logging guidance. Digital Security Act and PDA obligations apply
Are only the necessary data used, and is the quality sufficient?	GDPR (e.g., data minimization, data quality, anonymization); EHDS (e.g., quality criteria, provenance, interoperability); AI act requires representative and bias-assessed datasets	Reference the national data protection act, data quality, and registry rules; define roles for quality assessment (e.g., controller, HDAB, registry, and team members).	Norwegian practice relies on *NORMEN*, PDA, HReA registry governance, and national adoption of the EU AI act, emerging national health data service guidance on SPE requirements.
Where and how are analyses or AI models run?	AI sandbox, SPEs before CE & conformity assessment; AI act and MDR/IVDR regulate high-risk clinical AI development.	Identify national SPEs; high-risk AI development shall comply with the AI act and may be MDR/IVDR.	SPEs or authorized SAEs; national adoption of the EU AI act and sectoral AI guidelines
Who monitors compliance, and how?	GDPR (e.g., accountability, audit trails, and DPIAs); GDPR, NIS 2 incident reporting; EHDS requires SPE logging and audits; AI act requires post-market monitoring.	Specify national logging, audit, and incident obligations; identify responsible bodies (e.g., DPO, information and network security authority, supervisory authority).	PRA, and PRR impose logging for EHRs, SPEs and the national health data service logs, audits guided by *NORMEN*; DPO *(i.e., Datatilsynet)*, Norwegian Board of Health Supervision *(i.e., Helsetilsynet)* monitors.
How to disseminate results, close projects, and manage retention/reuse?	GDPR (e.g., storage limitation and research safeguards); EHDS (e.g., rules on output reuse and export); AI act (transparency and reporting)	National retention and archiving rules; national research and health data laws govern project closure.	Norwegian rules for reporting to ethics bodies and registries; HRA, HReA define retention; national practice on archiving or deletion (e.g., transfer to Health Archives, research data are deleted or anonymized unless REK approves longer retention).

## Discussion

Implementing lawful secondary use of health records extends beyond extracting a dataset and conducting analysis; it must comply with binding EU/EEA and national legislation. This requires coordinated legal, ethical, technical, and organizational decisions across the full project life cycle. As the guideline is intended to assist anyone planning secondary use, they must scope, classify, and operationalize secondary use projects before accessing the health records. The guideline above are based on practice and experience and incorporate EU regulations and obligations, such as EHDS, thereby supporting the transferability recommendation. For the guideline transferability to the EU/EEA, they should be interpreted in terms of functional equivalence rather than exact institutional replication. In other EU/EEA countries, the HDAB, ethics approval, and SAE/SPE functions may be distributed across national access bodies, accredited ethics committees, registry custodians, statistical offices, or institutional secure research environments. Users should therefore first map out who is responsible for ethics review, access authorization, secure processing, and compliance oversight at the national level, and then apply the nine steps accordingly. The nine-step guideline aims to operationalize lawful secondary use at the level of abstraction, jointly covering the legal, technical, and health care requirements discussed in the following paragraphs.

**Legal and ethical alignment** is a requirement to perform secondary use. We shaped the guideline by dual obligations under national law and EU/EEA legislation. The project classification step establishes lawful purposes, roles, and identifiability levels (e.g., non-anonymized, pseudonymized, or anonymized), while distinguishing Norway-specific and EU-wide requirements. Step 2 addresses legal basis and consent by combining confidentiality rules (HPA §21), exemptions, and research acts for conducting research. Consent under the HRA is not equivalent to consent under GDPR. There is a nuance in that, the HRA allows broad or ethics-approved consent, while GDPR requires specific, explicit, and revocable consent ^34^. For adolescents, provisions apply that require parental co-consent in specific contexts (HRA §17). This creates a challenge for researchers who must navigate parallel consent regimes and circumstances to determine when alternative legal bases apply to secondary-use processing rather than GDPR consent (e.g., exception vs public interest with national derogations).

The HDAB does not mention replacing national ethics committees; instead, the ethical review remains a separate national process. The current process, in which the regulatory ethics committee approves or provides an exemption upon ethics review, before access to the health record, contributes to procedural transparency and maintains trust. But researchers often report variation in ethical approval practices across regions, prolonged timelines^35^, and for multi-site projects, it can be a barrier. Within the EU, ethics committees remain nationally governed and not replaced by the EHDS; thus, projects must separately comply with both HDAB-managed data-access permits and national ethics regulatory approval. A shared library of ethical approval-vetted protocols and DPIA templates could reduce duplication and improve efficiency, but it requires national coordination and investment.

**Security, quality, and governance** is a second requirement for performing secondary use. Data access or data sharing agreements, security and privacy control ensure a secure infrastructure^15^. A secure analysis platform with logging, controlled access, and secure compute maintains auditable access logs, restrictions on downloading personal data align with EHDS requirements (EHDS Arts. 73(1e-f, 2))^3^. Member states may decide how to structure their HDAB, provided it has operational independence and sufficient administrative and technical capacity. This provides some governance freedom. The EHDS introduced HDAB-supervised secure processing environments for secondary use (i.e., SPE) should ensure export of only anonymized or statistical results and the structured handling of pseudonymized data (EHDS Art. 69, 73, 74(2))^3^. Our analysis shows overlaps between national SAE and EHDS-mandated SPE requirements. The Norwegian health information security and privacy guidelines (*i.e., NORMEN*) set baseline standards for information security, and the PDA reinforces GDPR provisions. Data governance, data quality, integrity, and consistency in practice can ensure that analyses are auditable and reproducible. While information security, quality, and governance enable secure and lawful secondary use, they are resource-intensive to operate. Researchers may experience a compliance overhead (e.g., data permit application to HDAB, DPIA, legal review, data-engineering effort) if secure platforms restrict methodological flexibility, which can slow experimentation or innovation. Without sufficient support, training, and funding, smaller research groups risk being disadvantaged compared to large, well-resourced institutions (e.g., in terms of compute, proprietary applications, domain experts)^19,25^.

**Patient, public engagement, and the protection of data-subject rights** is essential, as secondary use depends on sustained public trust^4,9,25,36^. GDPR rights of access, rectification, erasure, objection, opt-out/opt-in (EHDS Art. 71) and communication strategies continue to apply to personal data processing (GDPR Arts. 15 -18, 21)^37^. Under the EHDS, patients gain additional transparency regarding the right to view (e.g., patient portals or ‘MyHealth@EU’), know which entities accessed their records (EHDS Arts. 3 -8, 11)^21^. Those performing secondary use must therefore ensure clear information pathways, institutional webpages explaining the secondary use purpose, and mechanisms for handling data subject rights requests, even when data propagate into pseudonymized or anonymized datasets.

**Anonymization and pseudonymization** practices complement information security by keeping data safe for reuse while preserving its analytic utility. Fully anonymized data remain in scope for secondary use. Anonymization techniques (e.g., suppression, differential privacy, or aggregation) can be applied to reduce identifiability, but must be evaluated on a case-by-case basis, as neither the GDPR nor EHDS mentions the techniques. Pseudonymized data must remain inside SPE, with strict controls on re-identification risk, data-minimization, and output vetting. The guideline’s early classification step supports this distinction by requiring explicit assessment of the identifiability level based on identifiers, data sensitivity, context, and re-identification risk, and data minimization before data access requests.

**AI-specific considerations** are essential for secondary use as the AI systems make use of data. AI systems may be both tools and outputs for secondary use. The AI development lifecycle would span across all steps in the guidelines to complement oversight, governance, and model traceability. The AI act classifies many clinical AI systems with a risk of harm to safety, health, and human rights as high-risk. They require third-party conformity assessment, risk management, documentation, transparency, and post-market monitoring (AI Arts. 9-15, 72)^38^. Steps 6-9 of the guideline operationalize these duties through provenance tracking, technical documentation, data quality (e.g., accuracy, completeness, representativeness), human oversight, audit logs, compliance, and structured dissemination processes. In Norway, health records are analyzed within institutional infrastructure rather than on unsecured personal workstations^25^; it is already an established approach. The SPE and AI sandbox strengthen reproducibility and oversight but may again slow innovations and widen inequalities between large resourceful and smaller teams^19,25^, if adequate scientific freedom and functionalities are not provided within them.

**Continuous Learning (CL)** represents a systematic approach well suited to enabling sustainable, quality secondary use. This is mainly due to the inherently dynamic nature of health records, which are continuously expanding in volume, generated at high velocity, and increase in variety. This nature necessitates that analytics and AI models engage in CL. Unlike static one-time extracts, CL requires periodically retraining models with fresh data to maintain performance and correct biases from outdated datasets^39^. In the context of continuously updated EHRs, secondary use becomes an ongoing process rather than a single event. The EHDS requiring secondary-use processing in SPE with auditable access^3^, aligns well with the CL pipeline, which replaces one-time extracts with governed, repeatable ingestion (in batch or streaming), versioned data, and end-to-end lineage from raw sources through feature stores to model artifacts. CL also enables multi-type drift monitoring (data, covariate, label, concept) and explicit retraining triggers with staged rollout, consistent with literature on machine learning reliability and hidden technical debt^40^. Healthcare AI emphasizes that the temporal shift, meaning that a change in timeline or events occurring in chronological order or in multiple perspectives, is prevalent and that continuous monitoring and periodic updating are essential to have safe and effective AI system^39,41^. CL is discussed as an illustrative extension of the guideline, demonstrating that secondary-use governance must support not only one-time analyses but also iterative and longitudinal data reuse. The CL discussion exemplifies how the proposed nine-step guideline can remain applicable in dynamic, long-term, and real-time secondary-use scenarios.

As ‘HealthData@EU’ is rolled out later this decade, national HDAB and SPE technical specifications and accreditation (EHDS Art. 73(5), 75) are expected to be standardized across borders^8^. The applied CL must also respect the propagation of data-subject rights into caches, feature stores, and model artifacts^40,42^ and remain FAIR-aligned for stewardship and reproducibility^43^. A sustainable secondary use pipeline with CL would require cybersecurity with defense-in-depth, logging, repeatable ML extraction, and an automated data management pipeline^44^, requiring practices such as (i) support for event-driven or streaming ingestion, (ii) event or trigger based processing and data delivery, (iii) enforce schema or version control with backward-compatible change policies and change-data-capture^40^, (iv) capture metadata and lineage from raw sources through feature stores to model artifacts^40^, (v) implement reproducible feature engineering with code and data versioning and rollback strategies^40^, and (vi) define data-quality services (e.g., freshness, completeness, timeliness, and outlier bounds) with automated monitors and incident runbooks. While CL promises adaptability, it can introduce new risks, such as retraining cycles that can destabilize models, increase monitoring workload, and amplify hidden biases^39^. Moreover, the propagation of data-subject information into feature stores and model artifacts can raise compliance questions. Thus, while CL has the potential, its implementation would require sustained investment, national coordination, and clinical oversight.

**Strengths and limitations** are discussed here to clarify the study’s applicability. Our study complements innovation by focusing on regulatory development work. Unlike TEHDAS^1,2^ and TEHDAS2^31^, which focus on developing and piloting secondary use SPE infrastructures and cross-country coordination mechanisms, this study provides practice-oriented, role-aware, stepwise guidelines for navigating the secondary use of health records, covering binding and quasi-binding regulations at the EU and national levels, with Norway as a case. Applying a directed content analysis and legal expert review, the guideline synthesis is grounded in verifiable sources. Currently, both regulations and infrastructure remain in flux. Regulations relevant to secondary use, particularly the EHDS and the AI act, are undergoing phased application or national adaptation, and some interpretations may evolve as regulatory practice matures. Consequently, the guideline would require periodic revision to remain aligned with legal, technical, and operational updates. Nonetheless, providing a structured and cross-referenced guideline offers practical value during this transitional period. The methodology is practice-oriented and focuses on consolidating binding and quasi-binding regulatory requirements rather than a systematic review of all soft-law (non-legally binding) documents. Some context-specific practices may not be fully captured. Certain local or sector-specific arrangements, including public-private collaborations or commercial research partnerships, may introduce additional obligations that require clarification at the institutional level, and some data categories with dedicated regulatory regimes mentioned in scope above may require additional consideration and adaptation. Technical challenges common to secondary use, such as anonymization, handling derived or model-generated datasets (e.g., embeddings or feature vectors), ensuring data quality and interoperability across heterogeneous EHR systems (e.g., differing coding standards or incomplete records), and meeting security expectations for SPE require measures beyond regulatory guidelines alone. Finally, structured external validation of the guideline with researchers, regulators, patient groups, and industry stakeholders is planned for future work. Therefore, the guideline should be interpreted as a structured decision-support aid based on practical experience and regulations.

## Methods

We developed practice-oriented guideline by identifying and synthesizing binding and quasi-binding regulatory frameworks governing the secondary use of health records in the EU/EEA, and by drawing on practices and experience in Norway. To consolidate legal and regulatory requirements, we applied a directed content-analysis approach on sixteen documents (detail in Source identification below) using a structured extraction (Supplementary Table 2 and Supplementary Table 3) complemented by an expert-consensus based on the authors’ (ØN, TBR, PJT, HK) prior project experience. A practicing lawyer in health and AI (HK) supported legal interpretation to ensure accuracy regarding regulatory hierarchy, applicability, and binding force.

### Source identification

Source identification followed two strategies. First, author (DP) conducted iterative, targeted searches in the Norwegian legal database *‘Lovdata’*^45^ and the EU legal portal ‘EUR-Lex’^46^ using combinations of terms related to health records, secondary use, data protection, artificial intelligence, health research ethics, and information security (search summarized in Supplementary Table 1). This ensures coverage of binding European legislation and corresponding Norwegian acts and regulations. Second, senior authors (ØN, TBR, PJT), drawing on their experience in health data access, AI development, and regulatory compliance, reviewed the retrieved documents, and additionally included sector-specific codes of conduct *(NORMEN)*^32^ and ethical guidelines^36,47,48^. Lawyer (HK) was consulted to confirm their status, scope, and applicability.

Finally, a total of sixteen documents were included and identified (see Regulatory and legal context section), of which five are EU/EEA and one international source, seven Norwegian acts, and three Norway-specific guideline documents. Among the three guidelines, one is specific to health service providers, while the other two are ethical guidelines for science, technology, and big data research. All these are categorized as in Fig. 3.

### Eligibility criteria

Documents were included if they held binding or quasi-binding authority within the EU/EEA or expected to adopt in Norway; were in force or formally adopted by March 2025; directly governed or provided authoritative guidance for the secondary use of health records, healthcare data protection or information security, AI development or use in health settings, or health-research ethics and governance; and were publicly accessible in English or Norwegian. Documents focused exclusively on biological materials, non-health industrial data, or purely technical standards without regulatory authority were excluded.

### Regulatory and legal context

**Fig. 5:**
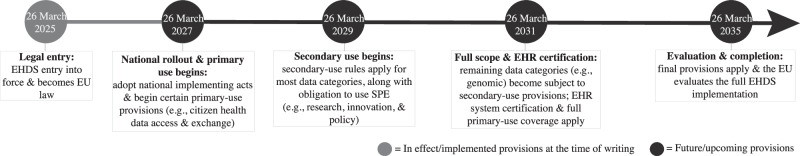
EHDS application timeline. Light gray circles indicate provisions already in effect at the time of writing, while dark circles indicate upcoming ones.

This section lists the sixteen documents that shaped the guideline. Figure 3 categorizes acts, laws, regulations, and guidelines considered in this study based on data protection, information security, health data frameworks, AI governance, ethics, and research integrity. The implementation of secondary use necessitates the integration of EU and national regulations such as those in Norway. It includes the following EU and international regulations:

a. EU European Health Data Space Regulation^3^: It entered into force in March 2025 as an EU regulation. The regulation is currently in a transition phase, pending full implementation as illustrated in Fig. 5, during which most obligations do not yet apply. For example, the mandatory use of SPEs will become applicable only when secondary-use provisions enter into force. Member states must adopt key provisions, such as those related to primary use namely the exchange of patient summaries and electronic prescriptions/dispensations, as well as for secondary use the technical requirements for SPE. In addition, member states must enable third countries and international organisations to join, ‘HealthData@EU’ by March 2034.

**Fig. 6:**
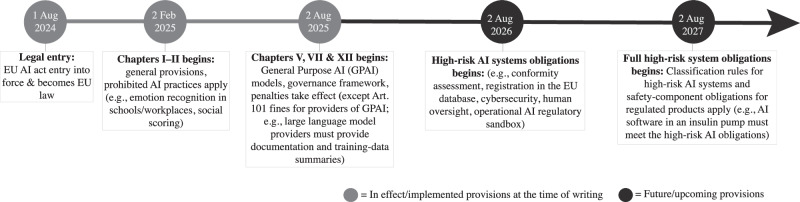
EU AI act application timeline. Light gray circles indicate provisions already in effect at the time of writing, while dark circles indicate upcoming ones.

During this period, member states are establishing the necessary governance structures and technical infrastructure to support the safe and effective flow of health records, while hospitals, IT vendors, and public bodies prepare for compliance. This transitional phase introduces uncertainty about operational responsibilities, which the guideline aims to clarify through steps and a checklist. This regulation will apply to Norway through the EEA Agreement^8^.

b. EU General Data Protection Regulation^37^: It is the core personal data protection and handling regulation in the EU/EEA, describing the lawful basis for processing, implementing information security measures, and the rights of the data subject (e.g., access, erasure, rectification). GDPR ensures that all processing of data follows the principles of data minimization, lawful processing, and protection of subject rights. In Norway, it is enacted through PDA^24^.

c. EU Artificial Intelligence Act^38^: It is the official harmonized rules for AI systems and services, including high-risk applications in healthcare within the EU/EEA. It guides the design and evaluation of AI models that utilize health records. Its hallmarks are transparency, risk management, human oversight, and benefitting from the potential of AIs. Figure 6 shows AI act application timeline. By 2 August 2026, every EU member state must have an AI sandbox. An AI sandbox is a supervised testing space overseen by a national authority, allowing companies to develop and test AI systems for a limited time in a controlled, real-world environment with regulatory guidance and safeguards (AI Act Art. 57). For example, a startup could pilot a hospital triage AI system, addressing any issues before going to market in an AI sandbox.

On June 30, 2025, the Norwegian government sent the Norwegian adaptation of the EU AI act, out for consultation^49^. As EU AI act will be adopted in Norway, it is relevant to consider when placing AI on the EU market or for forward compliance planning.

d. EU Medical Device Regulation^50^: It sets comprehensive requirements for the design, safety, performance, and post-market surveillance of medical devices. Covering provisions on medical device classification, data integrity, traceability, clinical evaluation, and continuous monitoring. These requirements are also relevant to the secondary use of health-related data generated by medical devices.

e. EU In Vitro Diagnostic Medical Device Regulation^51^: Specifically governing in vitro diagnostic technologies, establishing requirements for the safety, analytical performance, clinical evidence, and lifecycle management of diagnostic tests. Where data from in vitro diagnostics are reused for such as research, algorithm development, or analysis, organizations must follow IVDR.

f. World Medical Association Declaration of Helsinki^47^: International ethical guidelines for research involving human subjects. It reinforces ethical principles such as informed consent and minimization of harm throughout the research process.

Additionally, within the Norwegian national context, the following acts and regulations are central to secondary use:

a. Health Research Act^52^: Research using health records or performing medical or health quality improvement operations fall under it (HRA §9-12a). Such tasks require prior approval from the ethics committees, such as the Regional committees for medical and health research ethics (i.e., REK). The act defines criteria for consent, data handling, and project management. For health record secondary use in research, it necessitates obtaining ethical approval and detailing project management practices to ensure that secondary use is compliant.

b. Patient Records Act^23^: Outlines rules for handling health records, specifying secure data storage and access control, ensuring compliance with record-keeping standards, and sharing. It governs the use of EHR and the execution of secondary analysis.

c. Patient and User Rights Act^53^: Details patient rights and consent, including parental roles for minors under sixteen years old and partial consent rights for adolescents sixteen to eighteen years old. It underscores the significance of respecting children’s autonomy where appropriate. This act exerts a direct influence on consent processes, thereby ensuring that secondary use must respect patients’ rights and autonomy.

d. Health Personnel Act^54^: Regulates professional confidentiality and duty of care among healthcare personnel. It informs staff training, data handling procedures, and safeguards for sensitive information.

e. Patient Records Regulations^55^: Provides detailed record-keeping, technical, and organizational requirements for implementing PRA in practice. It operationalizes duties from the HPA (mainly §39, §40) and the PURA (on patient journal access). It turns those statutes into concrete requirements for journal content, logging, access control, and retention.

f. Health Register Act^56^: Sets the legal framework for creating and processing health registries outside direct patient care. Its purpose includes improving health, preventing disease, and enabling better services.

g. Personal Data Act^24^: Implements GDPR in Norway, mandating the lawful basis for personal data processing, data minimization, and transparency. It governs and guides data anonymisation and pseudonymization protocols to protect individual privacy.

h. Code of conduct for information security and data protection (NORMEN)^32^: It is a sector-specific guideline in Norway for healthcare institutions. It provides best practices on security, data classification, and breach management. It also underpins technical security measures (e.g., encryption and access control) and ensures data is classified correctly (e.g., confidential or strictly confidential).

i. The guidelines for research ethics in science and technology^48^ and Norwegian National Research Ethics - Big Data in Research^36^, offer detailed guidance on ethical research, especially concerning sensitive and big data studies. It is a benchmark for ethical data processing and model transparency, particularly in AI-driven analyses. The Norwegian Research Council has also established ethical standards for research, as outlined in the document entitled “Ethical Standards for Research”^57^.

### Synthesis

Our team, comprising legal practitioners and informaticians, conducted a directed content analysis of all sixteen documents iteratively. We organized extracted text into an extraction matrix aligned with nine predefined project-cycle domains (Steps 1-9), allowing deductive coding while permitting inductive subcoding where new themes emerged (Supplementary Table 2 and Supplementary Table 3). Through iterative consensus discussions, we synthesized, analyzed, and reviewed the extracted regulatory and ethical provisions and organized them into a nine-step operational guideline with concise and actionable checklist items and clickable references to regulations, supported by a source-to-guideline mapping (Supplementary Table 4). Authors with clinical-informatics and operational expertise refined the content to ensure its applicability to secondary use of health records, while legal counsel verified the accuracy of legal interpretation and regulatory hierarchy.

## Supplementary information

Supplementary Information

## Data Availability

No datasets were generated or analysed during the current study.

## References

[1] Abboud, L. et al. Report on secondary use of health data through european case studies. Tech. Rep. (Deliverable 5.1), Joint Action Towards the European Health Data Space (TEHDAS) https://tehdas.eu/app/uploads/2022/08/tehdas-report-on-secondary-use-of-health-data-through-european-case-studies-.pdf (2022). Updated version 30 August 2022; co-funded by the EU 3rd Health Programme, Grant Agreement No 101035467.

[2] European Commission, Directorate-General for Communications Networks, Content and Technology. 2024 digital decade ehealth indicator study. Tech. Rep. KK-05-24-386-EN-N, Publications Office of the European Union https://op.europa.eu/en/publication-detail/-/publication/c04f6162-3833-11ef-b441-01aa75ed71a1/language-en (2024). Report providing EU-wide monitoring of eHealth access as of 31 December 2023.

[3] Commission, E. Regulation on the european health data space https://eur-lex.europa.eu/legal-content/EN/TXT/?uri=OJ:L_202500327 (2022).Regulation of the European Parliament and of the Council.

[4] Safran, C. et al. Toward a national framework for the secondary use of health data: an american medical informatics association white paper. *Journal of the American Medical Informatics Association* **14**, 1–9 (2007). 17077452 10.1197/jamia.M2273 PMC2329823

[5] Montagu, D. The provision of private healthcare services in european countries: recent data and lessons for universal health coverage in other settings. *Frontiers in Public Health* **9**, 636750 (2021). 33791271 10.3389/fpubh.2021.636750 PMC8005513

[6] Kinge, J. M. et al. Disease-specific health spending by age, sex, and type of care in norway: a national health registry study. *BMC medicine* **21**, 201 (2023). 37277874 10.1186/s12916-023-02896-6 PMC10243068

[7] World Health Organization. Meeting on secondary use of health data. https://www.who.int/europe/news-room/events/item/2022/12/13/default-calendar/meeting-on-secondary-use-of-health-data (2022). “Secondary use of health data is the processing of health data for purposes other than the initial purposes for which the data were collected.”.

[8] Roberg, V. H. Enighet om helsedata (ehds). https://www.stortinget.no/no/Hva-skjer-pa-Stortinget/EU-EOS-informasjon/EU-EOS-nytt/2024/eueos-nytt---21.-mars-2024/enighet-om-helsedata-ehds/ (2024). (Accessed: 1 Jun, 2025) Library of Parliament: bibl@stortinget.no.Included in: EU/EEA news - 21 March 2024. Theme: Research, Healthcare, Human rights, Foreign affairs.

[9] Pant, D. et al. Secondary use of health records for prediction, detection, and treatment planning in the clinical decision support system: a systematic review. *BMC Medical Informatics and Decision Making* **25**, 1–13 (2025). 10.1186/s12911-025-03021-8 PMC12083156 40380138

[10] Helsedirektoratet. Nasjonal e-helsestrategi: Versjon 1.0. Government Report, Helsedirektoratet. https://www.helsedirektoratet.no/digitalisering-og-e-helse/nasjonal-e-helsestrategi/Nasjonal%20e-helsestrategi%20versjon%201.0.pdf/_/attachment/inline/a859bd1d-0615-4470-bc34-364dc0241a93:d93a1c54498c1dedec751e4dc4984e3c1150c939/Nasjonal%20e-helsestrategi%20versjon%201.0.pdf (2025). Page 16, line: “I dag genereres og samles det inn mye helsedata, men de utnyttes i begrenset grad.".

[11] Benson, T., Grieve, G. et al. *Principles of health interoperability: SNOMED CT, HL7 and FHIR*, vol. 3 (Springer, 2016).

[12] IHE International. Iti technical framework. Retrieved from https://www.ihe.net/resources/ (2025).

[13] Weiskopf, N. G. & Weng, C. Methods and dimensions of electronic health record data quality assessment: enabling reuse for clinical research. *Journal of the American Medical Informatics Association* **20**, 144–151 (2013). 22733976 10.1136/amiajnl-2011-000681 PMC3555312

[14] Kahn, M. G. et al. A harmonized data quality assessment terminology and framework for the secondary use of electronic health record data. *Egems* **4**, 1244 (2016). 27713905 10.13063/2327-9214.1244 PMC5051581

[15] World Health Organization. Cybersecurity and privacy maturity assessment and strengthening for digital health information systems https://www.who.int/europe/publications/i/item/WHO-EURO-2025-11827-51599-78854 (2025).

[16] Group, B. C. Norway health data leader digital. https://web-assets.bcg.com/82/2c/b38fc50f4b6786571bdae5d154b1/norway-health-data-leader-digital.pdf (2024) (Accessed: 24 March 2025).

[17] Norwegian Ministry of Health and Care Services. Forskrift om nasjonal løsning for tilgjengeliggjøring av helsedata. https://lovdata.no/dokument/SF/forskrift/2023-01-11-48.

[18] Recommendations for minimum requirements for secure processing environments in norway (2024). https://helsedata.no/contentassets/bdc68d971f1343ed92fb3d3673fb9905/helsedataradet/utkast---recommendations-for-minimum-requirements-for-secure-processing-environments-in-norway_29.11.2024-1.pdf#::text=data%20is%20sufficiently%20protected%20after,the%20national%20Health%20Data (Accessed: 24 March 2025).

[19] Lørup, L., Holgeid, K., Gray, A., Clawson, J. & Kamøy, S. Establishing Norway as a Health Data Leader: What Will It Take? https://web-assets.bcg.com/82/2c/b38fc50f4b6786571bdae5d154b1/norway-health-data-leader-digital.pdf (2024).

[20] The health industry https://www.regjeringen.no/en/dokumenter/the-health-industry/id2991874/?ch=4#::text=In%20recent%20years%2C%20changes%20have,further%20amendments%20to%20the%20regulations (2024).

[21] European Commission, Directorate-General for Health and Food Safety. European health data space summary. “European Health Data Space Regulation (EHDS)” - webpage https://health.ec.europa.eu/ehealth-digital-health-and-care/european-health-data-space-regulation-ehds_en.(2025). Published in the Official Journal of the European Union, L 63, on 5 March 2025; entered into force 26 March 2025.

[22] Government of Norway. Pasientjournalloven, lov-2014-06-20-42. Norwegian Law https://lovdata.no/lov/2014-06-20-42/S2. (2014).

[23] Government of Norway. Patient records act (pasientjournalloven, lov-2014-06-20-42). Norwegian Law (2014). National legislation.

[24] Government of Norway. Personal data act (personopplysningsloven, lov-2018-06-15-38). Norwegian Law (2018). National legislation.

[25] Shah, S. M. & Khan, R. A. Secondary use of electronic health record: Opportunities and challenges. *IEEE access* **8**, 136947–136965 (2020).

[26] Norwegian Ministry of Health and Care.Lov om humanmedisinsk bruk av bioteknologi m.m. (bioteknologiloven). Lov 5. desember 2003 nr. 100, Lovdata https://lovdata.no/lov/2003-12-05-100/S5-1. (2003).

[27] Norwegian Ministry of Health. Lov om behandlingsbiobanker (biobankloven). Lov 21. februar 2003 nr. 12, Lovdata https://lovdata.no/dokument/LTI/lov/2003-02-21-12 (2003).

[28] Norwegian Ministry of Health and Care. Lov om behandlingsbiobanker (behandlingsbiobankloven). Lov 21. februar 2003 nr. 12, Lovdata (NL) https://lovdata.no/dokument/NL/lov/2003-02-21-12 (2003).

[29] Riis, H. K. Health data in norway - status, challenges and international perspectives. https://www.uio.no/studier/emner/matnat/ifi/IN5090/v24/slides/2024-04-forelesning-ifi-eng.pdf (2024). Lecture slides for IN5090 (Digitalization in the Health Sector), University of Oslo. Lecture date: 30 April 2024.

[30] Helsedirektoratet. Regelverk om deling av helseopplysninger. Helsedirektoratet (nettressurs).https://www.helsedirektoratet.no/lov-og-forskrift/digital-deling-av-helseopplysninger/regelverk-om-deling-av-helseopplysninger. (Accessed: 18 August 2025).

[31] TEHDAS2 Coordination Team. Tehdas2 in brief. Online post. https://tehdas.eu/wp-content/uploads/2024/09/tehdas2-brief-leaflet-7.pdf. (Accessed: 17 Nov, 2025).

[32] Government of Norway. Code of conduct for information security and data protection (normen). Norwegian Standard https://www.helsedirektoratet.no/normen/norm-for-informasjonssikkerhet-og-personvern-i-helse-og-omsorgssektoren/om-normen/, (2022). Version 6.1, Version 7 (Accessed: 27 September 2025).

[33] Ministry of Health, Welfare and Sport (VWS), Netherlands. Programma HDAB-NL. https://www.datavoorgezondheid.nl/onderwerpen/h/health-data-access-body/programma (2023). Official Dutch government page on the HDAB-NL programme. Accessed 2026-04-20.

[34] Sikt - Norwegian Agency for Shared Services in Education and Research. Legal bases for personal data processing in research. https://sikt.no/en/tjenester/personverntjenester-forskning/personvernhandbok-forskning/legal-bases-personal-data-processing-research (2025). Accessed: 2025-09-19.

[35] De Lange, D. W. et al. Huge variation in obtaining ethical permission for a non-interventional observational study in europe. *BMC medical ethics* **20**, 39 (2019). 31159853 10.1186/s12910-019-0373-y PMC6547492

[36] Forskningsetikk. Big data in research: big opportunities, big challenges. Online. https://www.forskningsetikk.no/globalassets/dokumenter/4-publikasjoner-som-pdf/big-data-in-research---big-opportunities-big-challenges.pdf (2020). English translation, September 2021; Accessed: 18 February 2025.

[37] Union, E. Regulation (eu) 2016/679 of the european parliament and of the council of 27 april 2016 on the protection of natural persons with regard to the processing of personal data and on the free movement of such data, and repealing directive 95/46/ec (general data protection regulation). https://op.europa.eu/en/publication-detail/-/publication/3e485e15-11bd-11e6-ba9a-01aa75ed71a1/language-en (2016). Accessed: 2025-02-03.

[38] European Commission. Regulation (eu) 2024/1689 laying down harmonised rules on artificial intelligence. https://eur-lex.europa.eu/eli/reg/2024/1689/oj (2024). Accessed: 2025-02-03.

[39] Feng, J. et al. Clinical artificial intelligence quality improvement: towards continual monitoring and updating of ai algorithms in healthcare. *NPJ digital medicine* **5**, 66 (2022). 35641814 10.1038/s41746-022-00611-y PMC9156743

[40] Sculley, D. et al. Hidden technical debt in machine learning systems. *Advances in neural information processing systems* **28** (2015).

[41] Ghassemi, M., Oakden-Rayner, L. & Beam, A. The false hope of current approaches to explainable artificial intelligence in health care. *The Lancet. Digital health* **3 11**, e745–e750 (2021). 34711379 10.1016/S2589-7500(21)00208-9

[42] Herwanto, G. B., Ekaputra, F. J., Quirchmayr, G. & Tjoa, A. M. Toward a holistic privacy requirements engineering process: Insights from a systematic literature review. *IEEE Access* **12**, 47518–47542 (2024).

[43] Wilkinson, M. D. et al. The fair guiding principles for scientific data management and stewardship. *Scientific data* **3**, 1–9 (2016). 10.1038/sdata.2016.18 PMC4792175 26978244

[44] Polyzotis, N., Roy, S., Whang, S. E. & Zinkevich, M. A. Data management challenges in production machine learning. *Proc. 2017 ACM International Conference on Management of Data* https://api.semanticscholar.org/CorpusID:1925011 (2017).

[45] Lovdata Foundation. Lovdata - norsk. https://lovdata.no/ (1981). Accessed: 2025-11-16.

[46] Publications Office of the European Union. Eur-lex — access to european union law. https://eur-lex.europa.eu/homepage.html (1998). Accessed: 2025-11-12.

[47] WMA. Wma declaration of helsinki - ethical principles for medical research involving human participants. https://www.wma.net/policies-post/wma-declaration-of-helsinki/ (2024). Amended on 13th December 2024 by WMA.

[48] Forskningsetikk. Guidelines for research ethics in science and technology. Online https://www.forskningsetikk.no/globalassets/dokumenter/4-publikasjoner-som-pdf/guidelines-for-research-ethics-in-science-and-technology.pdf (2024). Accessed: 18 February 2025.

[49] Digitaliserings- og forvaltningsdepartementet. Høring - utkast til ny lov om kunstig intelligens (ki-loven) - gjennomføring av eus forordning om kunstig intelligens i norsk rett. https://www.regjeringen.no/no/dokumenter/3112327/id3112327/ (2025). Høring publisert 30.06.2025. Vår ref.: 25/2019.

[50] European Commission. Regulation (eu) 2017/745 of the european parliament and of the council on medical devices (medical device regulation, mdr). Official Journal of the European Union, L 117, 5 May 2017 https://eur-lex.europa.eu/legal-content/EN/TXT/?uri=CELEX:32017R0745 (2017).

[51] European Commission. Regulation (eu) 2017/746 of the european parliament and of the council on in vitro diagnostic medical devices (in vitro diagnostic regulation, ivdr). Official Journal of the European Union, L 117, 5 May 2017 https://eur-lex.europa.eu/legal-content/EN/TXT/?uri=CELEX:32017R0746 (2017).

[52] Government of Norway. Health research act (helseforskningsloven, lov-2008-06-20-44 §2). Norwegian Law (2008). National legislation.

[53] Government of Norway. Patient and user rights act (pasient- og brukerrettighetsloven, lov-1999-07-02-63). Norwegian Law (1999). National legislation.

[54] Government of Norway. Health personnel act (helsepersonelloven, lov-1999-07-02-64). Norwegian Law (1999). National legislation.

[55] Norwegian Ministry of Health and Care Services. Forskrift om pasientjournal (pasientjournalforskriften). https://lovdata.no/dokument/SF/forskrift/2019-03-01-168 (2019). Lovdata.

[56] Norwegian Ministry of Health and Care Services. Lov om helseregistre og behandling av helseopplysninger. Lovtidend (Norsk Lovtidend 2014, hefte 8, s. 1175) (2014). https://lovdata.no/dokument/NL/lov/2014-06-20-43 (Lov-2014-06-20-43, kunngjort 20. juni 2014, trådte i kraft 1. januar 2015).

[57] Norwegian Research Council.Ethical standards in research. Online post. https://www.forskningsradet.no/en/research-policy-strategy/ethical-standards/ (2019). (Accessed: 18 February 2025).

