# Supplementary material for: Guideline for secondary use of health records within Norwegian and EU regulatory frameworks
Source: NPJ Digit Med. 2026 May 28;9:532. doi: 10.1038/s41746-026-02784-2 (PMC13357775; doi:10.1038/s41746-026-02784-2)
Supplement: Supplementary file 1 — Supplementary Information [file 41746_2026_2784_MOESM1_ESM.pdf]

Supplementary Table 1: Overview of legal and regulatory source identification, search strategy, and corpus formation

(*We located, screened, and selected binding and quasi-binding regulatory, ethical, and legal documents relevant to the secondary use of health records in the EU/EEA and Norway. It includes search terms, databases, eligibility criteria, screening decisions, and the final corpus of 16 documents.)*

Component	Description
**Objective**	To identify binding and quasi-binding legal, regulatory, and ethical documents governing: (i) secondary use of health records and health data; (ii) data protection and information security; (iii) AI development and use in health settings; and (iv) health research ethics and governance, applicable to Norwegian health institutions in the EU/EEA context.
**Primary legal portals**	Lovdata (Norwegian laws and regulations); EUR-Lex (EU regulations and directives, including GDPR, EHDS,EU AI Act, MDR, IVDR); institutional sites (Norwegian Directorate of Health, Norway Data Protection Agency (*Datatilsynet*), national ethics committees).
**Search period**	Iterative searches from January 2025 to July 2025, with focused updates when the European Health Data Space Regulation (Regulation (EU) 2025/327) and the final text of the EU Artificial Intelligence Act became available.
**Search strings (Lovdata)**	Norwegian terms and combinations: "helseopplysninger", "pasientjournal", "helseregister", "sekundærbruk", "viderebehandling", "personopplysninger", "informasjonssikkerhet", "kunstig intelligens", "medisinsk utstyr", "in vitro diagnostikk", "forskningsetikk", "helseforskningsloven".
**Search strings (EUR-Lex and EU legal)**	English terms and combinations: "health data" AND "secondary use"; "electronic health records"; "European Health Data Space"; "secure processing environment"; "data permit"; "GDPR health"; "artificial intelligence act"; "high-risk AI systems"; "EU regulation medical devices"; "EU regulation in vitro diagnostic"; "research ethics guidelines".
**Additional identification (expert and hand-searching)**	Senior authors (ØN, TBR, PJT), with expertise in health data access, AI development, and regulatory compliance, reviewed the retrieved documents from the keyword search and additionally included Norwegian sectoral and ethics guidelines listed as numbered 6, 14, 15, and 16 in the final corpus of Supplementary Table 1 below.
**Inclusion criteria**	(i) Binding or quasi-binding authority within the EU/EEA or Norway; (ii) in force or formally adopted by March 2025; (iii) directly governing or providing authoritative guidance for secondary use of health records, health-data protection or information security, AI in health, or health-research ethics/governance; (iv) applicable or expected to be applicable to Norwegian health institutions; (v) publicly accessible in English or Norwegian.
**Exclusion criteria**	Documents focusing exclusively on biological materials without health-data governance; documents on non-health industrial data; purely technical standards (e.g., ISO) or norms without explicit regulatory or authoritative governance status.
**Screening and selection process**	(1) Title/summary screening of search results; (2) full-text review against domains and subdomains criteria below in Supplementary Table S2 ; (3) legal validation of relevance and authority by the health/AI lawyer (HK); (4) consensus confirmation within the author group through a meeting.
**Final corpus**	16 documents: (i) EU/EEA and international instruments (including GDPR, EHDS, EU AI Act, MDR, IVDR, and one international ethics framework); (ii) seven Norwegian acts and regulations directly affecting health-data use; (iii) three Norway-specific guideline/ethics documents. These are categorised by level and legal force in Figure 1 and the EU and others, and the Norwegian acts and regulations section of the article itself.<br>16 documents are:

	1. EU Artificial Intelligence Act<br>2. EU General Data Protection Regulation<br>3. EU European Health Data Space Regulation<br>4. EU Medical Device Regulations<br>5. EU In Vitro Diagnostics Medical Device Regulation<br>6. World Medical Association Declaration of Helsinki<br>7. *Helsepersonelloven (1999)* - Health Personnel Act (HPA)<br>8. *Pasient- og brukerrettighetsloven* - Patient and User Rights Act (PURA)<br>9. *Helseregisterloven (2014)* - Health Register Act (HReA)<br>10. *Pasientjournalforskriften (2019)* - Patient Journal Regulations<br>11. *Helseforskningsloven* (2008) - Health Research Act<br>12. *Pasientjournalloven* (2014*)*- Patient Record Regulation<br>13. *Personopplysningsloven* (2018) - Personal Data Act<br>14. Code of conduct for information security & data protection in the healthcare and care services sector *(Normen v7 and 6.1)*<br>15. Guidelines for research ethics in science and technology<br>16. National Research Ethics Committees (FEK)-Big Data in Research

Supplementary Table 2: Framework based on domains & subdomains

(Deductive coding method is used in the directed content analysis, structured according to the nine predefined guideline domains. Includes all subdomains and domain coding categories used to extract and classify regulatory provisions from the included documents after consensus among authors through discussion and meetings. DP conducted ten meetings with ØN, seven with TBR, three with HK, and two with PJT to reach expert consensus on domains.)

Domain 1: Project Classification

Subdomain	Description
1.1 Project type & scope	Determine if the project is health research, quality improvement, statistics, public interest, or other lawful category.
1.2 Roles & responsibilities	Controller/processor determination; joint controller arrangements.
1.3 Purpose specification	Required under GDPR Art. 5(1)(b), EHDS Art. 53
1.4 Data status	Personal data, special-category data; anonymised; pseudonymised.
1.5 Identifiability & pseudonymisation	GDPR Art. 4(5); Recital 26.
1.6 Registry-specific restrictions	HReA §§8–11.

Domain 2: Legal Basis and Consent

Subdomain	Description
2.1 GDPR legal bases	Art. 6(1)(e); 9(2)(h),(j).

2.2 National supplementary bases	HPA, PRA, other sector acts.
2.3 Confidentiality & dispensation	HPA §21, §29; HReA §19e.
2.4 Consent requirements	GDPR Art. 7, 13–14; HRA §13.
2.5 DPIA triggers	GDPR Art. 35 (linked to Step 5 for implementation).

Domain 3: Ethics and Regulatory Approval

Subdomain	Description
3.1 REK approval/exemption	HRA §9 for ethics committees (REK), §19 for application, and §33 is a requirement for prior approval.
3.2 Protocol & documentation	Requirements for protocol completeness.
3.3 Transparency/RoPA	GDPR Art. 30; Art. 13–14.

Domain 4: Data Access and Agreements

Subdomain	Description
4.1 Data sharing/user agreements	GDPR Art. 28(3); Art. 26.
4.2 SPE/SAE requirements	Second joint action Towards the European Health Data Space (TEHDAS-2) at EU, National SAEs (TSD, HUNT Cloud, SAFE).
4.3 Secure transfer	Encryption, access control, secure channels.
4.4 Registry access	HReA §19e–f (dispensation, access windows).
4.5 EHDS data permit	Legal requirement for secondary use under EHDS Art. 68, and EHDS Arts. 55, 67 health data access bodies
4.6 EHDS secure processing environment (SPE)	EHDS Art. 73 and Chapter IV on SPE operation by health data access bodies.

Domain 5: Data Security and Privacy

Subdomain	Description
5.1 Security of processing	GDPR Art. 32; PRA §22; PRR §14; EHDS Arts. 57, 73
5.2 Encryption & key management	GDPR Art. 32.
5.3 Access control, MFA, RBAC	Sectoral norms & GDPR.

5.4 Logging & log review	PRR §14; Normen 5.4.4.
5.5 Breach notification	GDPR Art. 33–34.
5.6 International transfers	GDPR Arts. 44–49. EHDS Art. 88, 90
5.7 Vendor assessment	GDPR Art. 28.
5.8 EU NIS2 obligations	Cybersecurity + incident reporting for essential/important entities.

Domain 6: Data Minimisation & Quality

Subdomain	Description
6.1 Data-field justification	Necessity & proportionality.
6.2 Free-text sweeps	Prevent unintended PII extraction.
6.3 Quality checks	Accuracy principle (GDPR Art. 5(1)(d)).

Domain 7: Analysis and AI Development

Subdomain	Description
7.1 Validated tools	Compliance with SPE/SAE constraints.
7.2 Re-identification prohibition	Unless authorised.
7.3 Preprocessing documentation	GDPR Art. 5(2); AI Act Art. 10.
7.4 AI Act classification (high-risk AI)	AI Act Arts. 6–7; Annex III.
7.5 AI Act obligations	Risk management (Art. 9), data governance (Art. 10), technical documentation (Art. 11), record keeping (Art. 12), transparency (Art. 13), human oversight (Art. 14), accuracy & cybersecurity (Art. 15).
7.6 MDR/IVDR qualification	Software as medical device or IVD.
7.7 Post-market surveillance (MDR/IVDR)	Continuous monitoring obligations.

Domain 8: Compliance Monitoring and Auditing

Subdomain	Description
8.1 Internal/external audits	GDPR Art. 24.
8.2 Access-log review	PRA §22; PRR §14.

8.3 Purpose adherence	GDPR Art. 5(1)(b).
8.4 Amendment procedures	HRA §33; GDPR accountability.

Domain 9: Dissemination, Close-Out, Retention, Deletion

Subdomain	Description
9.1 Anonymisation checks	GDPR Art. 89(1).
9.2 Reporting (REK, funders)	HRA §33.
9.3 Retention limits	GDPR Art. 5(1)(e); HReA §19f.
9.4 Data deletion/anonymisation	Documentation for accountability.
9.5 Archival of documentation	Non-identifiable documents only.
9.6 AI lifecycle monitoring	AI Act; MDR/IVDR PMS requirements.

Supplementary Table 3: Extraction matrix used for directed content analysis and above domain, subdomain-based synthesis of regulatory, ethical, and legal sources into the nine-step guideline

(Provides the extraction matrix used to document all regulatory, ethical, and legal provisions, coded from the included sources, with links to guideline steps, subdomains, and operational implications. Each row demonstrates how a given provision was interpreted and mapped into the final nine-step guideline.)

Document	Jurisdiction	Legal status	Article / Section	Extracted provision (summary)	Guideline Step (1–9)	Subdomain code (from Supp. 2A)	Operational implication/checklist cue	Notes
EU-GDPR	EU/EEA	Binding regulation	Art. 4, Art. 4(7), Art. 5(1)(b)	Defines “controller” and “processor”; requires processing to have specified, explicit, and legitimate purposes.	1 – Project classification	1.2 Roles & responsibilities; 1.3 Purpose specification	At project classification, document the project’s purpose and identify all controller(s) and processor(s) with their roles.	Core EU-level definitions; applies to all personal-data processing.
EU-GDPR	EU/EEA	Binding regulation	Art. 4(1), Art. 4(5), Art. 9(1); Recital 26	Defines personal data, pseudonymisation, special	1 – Project classification	1.4 Data status; 1.5 Identifiability &	Classify the dataset as personal, special-category, or	EU-level definition of data status; national law builds on these concepts.

				categories of data, and clarifies criteria for anonymisation; pseudonymised data remain personal data.		pseudonymisation	anonymised; treat pseudonymised health data as personal data and handle accordingly.	
NO-HReA (Health Registry Act)	Norway	Binding act	§§ 8–11	Regulates establishment and use of health registries; sets conditions, purposes, and restrictions for registry data, including secondary use.	1 – Project classification	1.6 Registry-specific restrictions	When using health registries, document registry-specific legal restrictions and ensure the project purpose is compatible with registry terms.	National act specifying registry conditions; applies when registries are used.
NO-HRA (Health Research Act)	Norway	Binding act	§2, §4	Defines “medical and health research” and scope of the act, including which projects fall under REK review.	1 – Project classification	1.1 Project type & scope	Determine whether the project is medical/health research; if so, classify under the Health Research Act and REK competence.	National classification of health-research pathway.
EU-GDPR	EU/EEA	Binding regulation	Art. 6(1)(e)	Permits processing necessary for the performance of a task carried out in the public interest or in the exercise of official authority.	2 – Legal basis & consent	2.1 GDPR legal bases	For public-sector bodies, determine whether secondary use can rely on Art. 6(1)(e) as a lawful basis and document this in the project record.	Core lawful basis for many public-sector projects.
EU-GDPR	EU/EEA	Binding regulation	Art. 9(2)(h),(j); Art. 89(1)	Permits processing of special-category health	2 – Legal basis & consent	2.1 GDPR legal bases	Where special-category health data are processed,	Governs lawful use of health data for research/management/statistics.

				data for healthcare, management, and research/statistics with appropriate safeguards, including data minimisation and pseudonymisation.			document reliance on Art. 9(2)(h) or (j) and the safeguards (e.g., pseudonymisation) in place.	
NO-HPA (Health Personnel Act)	Norway	Binding act	§21, §29	Establishes duty of confidentiality for health personnel and conditions under which dispensation from confidentiality can be granted, including for research/statistics in the public interest.	2 – Legal basis & consent	2.3 Confidentiality & dispensation	Verify whether project requires dispensation from confidentiality and, if so, obtain and document the dispensation decision before data use.	National confidentiality rules; complement GDPR.
NO-HeRA(Health Registry Act)	Norway	Binding act	§19e	Provides mechanisms for dispensation from confidentiality for use of health registry data for research and statistics in the public interest.	2 – Legal basis & consent	2.3 Confidentiality & dispensation	For registry-based projects, assess and document need for dispensation under HReA §19e as part of the legal basis.	Specific to registry data and research/statistics use.
NO-PRA (Patient Record Regulation)	Norway	Binding act	Relevant sections on processing and confidentiality	Governs processing of information by public authorities, including confidentiality and administrativ	2 – Legal basis & consent	2.2 National supplementary bases	Where public authorities act as controllers, identify and record relevant PRA provisions that complement	Provides an additional national legal basis for public-sector processing.

				e decision-making requirements.			GDPR Art. 6(1)(e).	
EU-GDPR	EU/EEA	Binding regulation	Art. 7, Arts. 13–14	Sets conditions for valid consent and information duties when consent is used as lawful basis.	2 – Legal basis & consent	2.4 Consent requirements	Where consent is used, ensure that information provided and documentation meet GDPR consent and transparency requirements.	Applies when consent is chosen as lawful basis.
NO-HRA	Norway	Binding act	§13	Sets specific requirements for consent in medical and health research, including documentation and withdrawal.	2 – Legal basis & consent	2.4 Consent requirements	Harmonise GDPR consent with HRA §13; ensure research consent forms cover secondary use or re-consent as required.	Applies to health research classified under HRA.
EU-GDPR	EU/EEA	Binding regulation	Art. 35	Requires a Data Protection Impact Assessment (DPIA) where processing is likely to result in a high risk to rights and freedoms, including certain health and AI uses.	2 & 5 – Legal basis; security	2.5 DPIA triggers; 5.1 Security of processing	Assess whether the project requires a DPIA; if so, complete it before accessing the data and document outcomes.	DPIA often mandatory for large-scale secondary-use projects.
NO-HRA	Norway	Binding act	§9, §10, §33	Requires prior approval or exemption by REK for medical and health research using health records and sets conditions for continued	3 – Ethics & regulatory approval	3.1 REK approval/exemption; 8.4 Amendment procedures	Obtain REK approval or documented exemption before data access; seek amendments if project scope changes.	National ethics and research-governance requirement.

				use and changes.				
EU-GDPR	EU/EEA	Binding regulation	Art. 30; Arts. 13–14	Requires controllers/processors to maintain a Record of Processing Activities and provide transparency information to data subjects.	3 – Ethics & regulatory approval	3.2 Protocol & documentation; 3.3 Transparency/RoPA	Prepare or update RoPA entries and transparency notices for the project before or at the start of processing.	Supports accountability and transparency obligations.
EU-GDPR	EU/EEA	Binding regulation	Art. 28(3); Art. 26	Requires data processing agreements with processors and written arrangements for joint controllers, including allocation of responsibilities and safeguards.	4 – Data access & agreements	4.1 Data sharing/user agreements	Before data transfer, ensure DPAs/DSAs with controllers/processors are in place specifying purpose, security, retention, and responsibilities.	Key contractual mechanisms in multi-party projects.
EU-GDPR	EU/EEA	Binding regulation	Art. 32	Requires appropriate technical and organisational measures (e.g., encryption, access control, logging) for security of processing.	4 & 5 – Data access; security	4.2 SPE/SAE requirements; 5.1 Security of processing	Use secure processing environments (SPE/SAE) with encryption, role-based access and logging for secondary-use health data projects.	Applies to all high-risk health-data processing.
EU-EHDS (European Health Data Space Regulation)	EU/EEA	Binding regulation	Provisions on data permits for secondary use (e.g., Art. 68) and secure processing environments (e.g., Arts. 73–75), and	Requires that secondary use of electronic health data occurs under a data permit and within a secure processing environment managed by a	4 & 5 – Data access; security	4.5 EHDS data permit; 4.6 EHDS SPE	Where EHDS applies, obtain a data permit for secondary use and conduct all processing within an EHDS-compliant secure processing environment	Adds EU-level conditions for cross-border secondary use of health data.

			permitted purposes (e.g., Art. 53)	health data access body, for specified permitted purposes.			for allowed purposes only.	
NO-HReA	Norway	Binding act	§19f	Regulates storage limitation and retention periods for health registry data.	4 & 9 – Access; close-out	4.4 Registry access; 9.3 Retention limits	Determine and document the legally permitted retention period and enforce deletion/anonymisation at the end of that period.	Applies specifically to health registries in Norway.
Normen (Information security, privacy norm for the health sector)	Norway	Quasi-binding sector norm	Sections on access control, logging, encryption, SPE usage (e.g., sections 5.2, 5.3.5, 5.2.1, 5.4.4)	Provides sector-specific guidance on access control, encryption, logging, and secure environments for health-data processing.	5 – Data security & privacy	5.2 Encryption & key management; 5.3 Access control; 5.4 Logging & log review	Implement access control, encryption and logging in line with Normen for health-sector SPE/SAE environments.	Widely adopted norm; complements binding GDPR and national rules.
NO-PRA; NO-PRR	Norway	Binding regulations	PRA §22; PRR §14	Set requirements for logging access to health systems and performing periodic log review.	5 & 8 – Security; auditing	5.4 Logging & log review; 8.2 Access-log review	Log all data extraction and access from EHR/registry systems and perform periodic, risk-based log review.	National logging and review obligations.
EU-GDPR	EU/EEA	Binding regulation	Arts. 44–49	Regulates international transfers of personal data outside the EEA, including adequacy, standard contractual clauses, and derogations.	5 – Data security & privacy	5.6 International transfers	Where personal data are transferred outside the EEA, choose and document a lawful transfer mechanism and perform a transfer risk assessment.	Essential for cross-border collaborations beyond EEA.

EU NIS2 Directive	EU/EEA	Binding directive (requires national implementation)	General provisions on cybersecurity and incident reporting for essential and important entities	Imposes cybersecurity and incident reporting obligations on covered entities, including many health providers and digital infrastructures.	5 – Data security & privacy	5.8 NIS2 obligations	Where organisations fall under NIS2, align SPE/SAE governance and incident handling with NIS2 requirements and national implementation.	Applies to entities classified as essential or important under NIS2.
EU-GDPR	EU/EEA	Binding regulation	Art. 5(1)(b),(c)	Establishes purpose limitation and data minimisation principles.	6 – Minimisation & quality	6.1 Data-field justification	When extracting data, include only fields strictly necessary for the approved objectives and document justification.	Applies to all secondary-use datasets.
EU-GDPR	EU/EEA	Binding regulation	Art. 5(1)(d)	Requires data to be accurate and, where necessary, kept up-to-date.	6 – Minimisation & quality	6.3 Quality checks	Implement periodic data quality checks (e.g., completeness, plausibility) in line with accuracy obligations.	Supports quality governance in secondary-use projects.
EU-GDPR	EU/EEA	Binding regulation	Art. 25	Embeds data protection by design and by default, including minimisation and access controls.	6 – Minimisation & quality	6.2 Free-text sweeps	Conduct post-extraction sweeps for unexpected/disallowed content (e.g., free text containing identifiers) to uphold data protection by design and default.	Operationalises minimisation in derived datasets.
EU-AI Act	EU/EEA	Binding regulation	Arts. 6–7; Annex III	Establishes criteria and categories for high-risk AI systems, including many AI uses	7 – Analysis & AI development	7.4 AI Act classification (high-risk AI)	For AI components, perform and document an AI risk classification to determine	Determines applicability of high-risk obligations.

				in healthcare and patient care decisions.			whether the system is high-risk under the AI Act.	
EU-AI Act	EU/EEA	Binding regulation	Arts. 9–10	Requires risk management and data/data-governance measures for high-risk AI, including data representativeness, relevance, and bias assessment.	7 – Analysis & AI development	7.5 AI Act obligations (risk management & data governance)	For high-risk AI, implement a documented risk management process and data governance, including bias assessment and mitigation.	Complements GDPR DPIA for AI.
EU-AI Act	EU/EEA	Binding regulation	Arts. 11–12	Requires comprehensive technical documentation and record-keeping for high-risk AI systems.	7 – Analysis & AI development	7.5 AI Act obligations (documentation & record-keeping)	Maintain technical documentation and records (e.g., model description, training data sources, intended purpose) for AI systems used in the project.	Aligns with transparency and accountability principles.
EU-AI Act	EU/EEA	Binding regulation	Arts. 13–14	Requires appropriate transparency and human oversight measures for high-risk AI systems.	7 – Analysis & AI development	7.5 AI Act obligations (transparency & oversight)	Ensure that AI outputs are explainable to users and that human oversight and intervention mechanisms are defined and documented.	Particularly relevant for clinical settings.
EU-AI Act	EU/EEA	Binding regulation	Art. 15	Requires accuracy, robustness, and cybersecurity for high-risk AI systems.	7 – Analysis & AI development	7.5 AI Act obligations (accuracy & cybersecurity)	Implement technical and organisational measures to maintain AI accuracy, robustness, and cybersecurity, and document how these are	Links AI properties to broader cybersecurity regime.

							monitored over time.	
EU-MDR (Regulation (EU) 2017/745)	EU/EEA	Binding regulation	Provisions on qualification of software as medical device, clinical evaluation, and post-market surveillance	Requires that software meeting the definition of a medical device undergo appropriate conformity assessment, clinical evaluation, and post-market surveillance.	7 & 9 – Analysis; close-out	7.6 MDR/IVDR qualification; 7.7 Post-market surveillance (PMS)	If AI/software qualifies as a medical device, ensure MDR-conforming qualification, evaluation, and PMS are planned and documented.	Adds device-regulatory obligations beyond data protection.
EU-IVDR (Regulation (EU) 2017/746)	EU/EEA	Binding regulation	Provisions on in vitro diagnostic devices, including software and PMS	Requires classification, performance evaluation and ongoing surveillance for in vitro diagnostic devices, including software.	7 & 9 – Analysis; close-out	7.6 MDR/IVDR qualification; 7.7 Post-market surveillance (PMS)	Where AI/software functions as an IVD, ensure IVDR requirements are addressed alongside data-protection obligations.	Relevant for diagnostic decision-support use cases.
EU-GDPR	EU/EEA	Binding regulation	Art. 5(1)(e); Art. 5(2)	Establishes storage limitation and accountability principles, requiring that personal data not be kept longer than necessary and that controllers can demonstrate compliance.	9 – Dissemination, close-out, retention & deletion	9.3 Retention limits; 9.4 Data deletion/anonymisation	At project closure, irreversibly delete or anonymise personal data and document the method and date of deletion/anonymisation for accountability.	Fundamental to end-of-life data governance.
NO-HReA	Norway	Binding act	§19f	Specifies retention periods and conditions for continued storage of health registry data.	9 – Dissemination, close-out, retention & deletion	9.3 Retention limits	Align project retention and deletion plans with HReA §19f for registry data.	Registry-specific retention requirement.

NO-HRA; ethics guidelines	Norway / International	Binding act / quasi-binding ethics frameworks	HRA reporting requirements; national ethics guidelines for research in science/technology	Require reporting to REK and funders, inclusion of ethics statements in publications, and adherence to recognised research ethics principles.	9 – Dissemination, close-out, retention & deletion	9.2 Reporting (REK, funders); 9.1 Anonymisation checks	Include ethics and GDPR statements in dissemination, report progress/final results to REK and funders, and ensure outputs are anonymised.	Ensures ethical and regulatory transparency at dissemination.
EU-AI Act; EU-MDR /IVDR	EU/EEA	Binding regulations	Lifecycle and PMS obligations for AI systems and medical/IVD devices	Require ongoing monitoring, incident handling, and, where relevant, updates to conformity assessments throughout the lifecycle of AI systems and medical/IVD devices.	9 – Dissemination, close-out, retention & deletion	9.6 AI lifecycle monitoring	Where AI systems continue in clinical use beyond the project, ensure operational governance complies with AI Act and MDR/IVDR lifecycle requirements, including PMS and incident reporting.	Relevant when project outputs lead to or support deployed AI medical products.

Supplementary Table 4: Mapping of Guideline Checklist Items to Legal/Regulatory Provisions

(The linkage between each checklist item in the nine-step guideline and the corresponding legal, regulatory, or ethical provisions from EU-level and national instruments. It shows the analytical chain from source document to concept to guideline item.)

Step	Checklist item (short form)	Main source(s)	Interpretation / mapping notes
**Project classification**	Classify project type and document purpose, controller(s), processor(s), datasets and roles.	GDPR Art. 4, 4(7), 5(1)(b); HRA §2, §4	GDPR defines “controller”, “processor” and purpose limitation; HRA clarifies when activities qualify as health research under REK.
	Identify data status (personal vs special-category vs anonymised); treat pseudonymised data as personal.	GDPR Art. 4(1), 4(5), 9(1); Recital 26; HReA §2f	EU definitions are primary; national law clarifies pseudonymised registry data.

	Record registry-specific restrictions when using health registers.	HReA §§ 8–11	Governs creation and use of health registries and sets constraints on reuse.
**2 – Legal basis and consent**	Determine and document GDPR legal basis (Art. 6) and special-category basis (Art. 9), including public-interest and research/statistics bases with safeguards.	GDPR Art. 6(1)(e); 9(2)(h),(j); 89(1)	Establishes EU-level lawful bases and safeguards for secondary uses.
	Identify and document supplementary sector-specific legal bases, where required (e.g. health personnel, public administration).	HPA; PRA	National acts provide complementary legal authority in specific sectors.
	Conduct DPIA where processing is likely to result in high risk or document rationale if not needed.	GDPR Art. 35	DPIA is mandatory for many large-scale or high-risk health-data uses, including some AI cases.
	Ensure confidentiality obligations are met or dispensation obtained for research/statistics when justified.	HPA §21, §29; HReA §19e	National confidentiality rules and dispensation mechanisms apply alongside GDPR.
	When using consent, ensure it is valid under GDPR and HRA and covers secondary use or re-consent as needed.	GDPR Arts. 7, 13–14; HRA §13	Combines EU consent requirements with research-specific national rules.
**3 – Ethics and regulatory approval**	Obtain REK approval or formal exemption before accessing health records for research.	HRA §9, §33	REK is the competent ethics/regulatory body for health research projects using health records.
	Prepare RoPA and transparency notices to comply with data protection requirements.	GDPR Arts. 30, 13–14	Ensures documentation and transparency obligations are addressed early.
**4 – Data access and agreements**	Secure data-sharing/user agreements and data-processing agreements specifying purpose, scope, duration, security, and destruction/return.	GDPR Art. 28(3), 26	Contractual instruments operationalise controller–processor and joint-controller responsibilities.
	Ensure use of secure processing environments (SPE/SAE) for analysis and development, with appropriate technical and organisational safeguards.	GDPR Art. 32; national norms (e.g. Normen); institutional SAE/SPE guidance	SPE/SAE is the implementation of “appropriate technical and organisational measures” for high-risk health data processing.
	Where data fall under the EHDS, obtain a data permit for secondary use and ensure access via an EHDS-compliant secure processing environment.	EHDS Regulation (EU 2025/327), provisions on data permits and secure processing environments (e.g. sections on “data permit for secondary use of electronic health data” and “secure processing environment”).	EHDS adds a harmonised EU-level mechanism for secondary use and mandates SPEs operated by health data access bodies.

	Determine maximum permitted retention and access windows and record them in agreements and project documentation.	HReA §19f; registry access conditions	National registry law sets retention/access windows for registry data; agreements should mirror these.
**5 – Data security and privacy**	Implement defence-in-depth security: encryption at rest/in transit, role-based access control, MFA, logging, risk-based log review.	GDPR Art. 32; PRA §22; PRR §14; Normen	Combined EU and Norwegian security and logging obligations, operationalised in SPE/SAE.
	Ensure DPIA is completed before access/analysis when required (e.g. large-scale health records, innovative AI, vulnerable groups).	GDPR Art. 35	Links earlier DPIA decision (Step 2) to concrete implementation before data access.
	If transferring personal data outside the EEA, verify legal basis and mechanism (adequacy, SCCs, derogations), assess third-country laws and protections, and document a transfer impact assessment.	GDPR Arts. 44–49; Datatilsynet guidance	Ensures cross-border transfers meet EU and Norwegian supervisory expectations.
	Vet third-party tools/vendors and sign appropriate processor agreements.	GDPR Art. 28	Extends controller obligations to any third-party services used.
**6 – Data minimisation and quality**	Extract only fields that are necessary and approved, avoiding excessive or unrelated personal data in primary and derived datasets.	GDPR Art. 5(1)(b),(c)	Applies purpose limitation and data minimisation.
	Perform post-extraction sweeps for unexpected/disallowed content (e.g. free text with identifiers, images) unless explicitly approved.	GDPR Art. 5(1)(c); Art. 25	Ensures minimisation is maintained after extraction.
	Set periodic data quality reviews aligned with project objectives and evolving standards/regulations.	GDPR Art. 5(1)(d),(2)	Accuracy and accountability principles justify ongoing quality review.
**7 – Analysis and AI development**	Use validated analytical tools and methods that comply with SPE/SAE requirements; do not attempt re-identification unless explicitly authorised.	GDPR Art. 5(1)(a),(b); security principles	Ensures analysis respects original purposes and security conditions.
	Maintain comprehensive preprocessing documentation (cleaning, transformation, anonymisation), and technical documentation to support transparency and reproducibility.	AI Act Art. 10; AI Act Annexes on technical documentation; GDPR Art. 5(2)	Aligns AI and data protection documentation requirements.
	Perform AI risk classification and document whether the system is high-risk within the meaning of the EU AI Act.	AI Act Arts. 6–7; Annex III	Classification step determines whether high-risk obligations apply.
	For high-risk AI, implement risk management, data governance, technical documentation, record-keeping, transparency, human oversight and accuracy/cybersecurity controls.	AI Act Arts. 9–15	These provisions specify the mandatory control set for high-risk AI systems.

	Where AI qualifies as a medical device or IVD, ensure MDR/IVDR-compliant qualification, clinical evaluation, and post-market surveillance planning.	MDR (Regulation (EU) 2017/745); IVDR (Regulation (EU) 2017/746), including PMS provisions	MDR/IVDR overlay applies when secondary-use data supports or is embedded in software that is a medical device/IVD.
**8 – Compliance monitoring and auditing**	Conduct internal audits (and facilitate external audits) to detect and correct compliance issues across the project lifecycle.	GDPR Art. 5(2), 24; PRA §22; PRR §14	Translates accountability and controller responsibility into ongoing audit.
	Log all data extraction and access to source systems and perform periodic log review with a risk-based cadence.	PRA §22; PRR §14; Normen	Responds to Norwegian logging requirements and good practice guidance.
	Ensure data are used only for the approved protocol and seek amendments before new purposes or analyses.	GDPR Art. 5(1)(b); HRA §33	Protects against scope creep and unlawful purpose expansion.
**9 – Dissemination, close-out, retention, deletion**	Before releasing results, ensure that no individual can be re-identified from outputs (e.g. via small cell counts, indirect identifiers).	GDPR Art. 5(1)(c); 89(1)	Justifies robust anonymisation and disclosure control.
	Include required ethics and data-protection statements in publications and reports (e.g. REK reference, GDPR compliance).	HRA; ethics guidelines	Standard practice in research ethics and reporting.
	At project close-out, irreversibly delete or anonymise personal data and double-check backups and secondary copies; document method and date.	GDPR Art. 5(1)(e),(2); HReA §19f	Operationalises storage limitation and accountability.
	Archive key documentation (approvals, consent forms, analysis scripts, final reports, DPIA) without identifiable raw data for the period required by institutional policy.	GDPR Art. 5(1)(e); 89(1); institutional guidance	Retention of non-identifiable documentation is distinct from data retention.
	Where data are retained or reused, record new legal basis (e.g. renewed consent, extended REK approval, transfer to lawful data bank/registry/biobank).	GDPR Art. 6(1), 9(2); HRA §§ 13–14, 33; health registry law	Ensures renewed lawful basis for any extended use beyond original approval.
	If an AI system continues in clinical use, govern it under operational protocols and ensure ongoing compliance with AI Act, MDR/IVDR post-market surveillance and monitoring requirements.	AI Act lifecycle obligations; MDR/IVDR PMS provisions	Integrates data-use governance with lifecycle oversight of AI-based medical devices/IVDs after deployment.
